# IGF2BP2-induced circRUNX1 facilitates the growth and metastasis of esophageal squamous cell carcinoma through miR-449b-5p/FOXP3 axis

**DOI:** 10.1186/s13046-022-02550-8

**Published:** 2022-12-15

**Authors:** Chang Wang, Mingxia Zhou, Peiyu Zhu, Chenxi Ju, Jinxiu Sheng, Dan Du, Junhu Wan, Huiqing Yin, Yurong Xing, Hongle Li, Jing He, Fucheng He

**Affiliations:** 1 Department of Medical Laboratory, The First Affiliated Hospital of Zhengzhou University, Zhengzhou, 450052 China; 2 Department of Gastroenterology, The First Affiliated Hospital of Zhengzhou University, Zhengzhou, 450052 China; 3 Department of Biomedical Informatics, School of Basic Medical Sciences, Peking University Health Science Center, Beijing, 100191 China; 4 Center of Health Examination, The First Affiliated Hospital of Zhengzhou University, Zhengzhou, 450052 China; 5 Department of Molecular Pathology, The Affiliated Cancer Hospital of Zhengzhou University, Zhengzhou, 450008 Henan China; 6 Department of Breast Surgery, The First Affiliated Hospital of Zhengzhou University, Zhengzhou, 450052 China

**Keywords:** Esophageal squamous cell carcinoma (ESCC), CircRUNX1, FOXP3, IGF2BP2, Biomarker

## Abstract

**Background:**

Esophageal squamous cell carcinoma (ESCC) is one of the most common digestive malignancies with relatively high morbidity and mortality. Emerging evidence suggests circular RNAs (circRNAs) play critical roles in tumor cell malignancy. However, the biological function and clinical significance of many circRNAs in ESCC remain elusive.

**Methods:**

The expression level and clinical implication of circRUNX1 in ESCC tissues were evaluated using qRT-PCR. In vitro and in vivo functional studies were conducted to investigate the underlying biological effects of circRUNX1 on ESCC cell growth and metastasis. Moreover, bioinformatics analysis, RNA sequencing (RNA-seq), RNA immunoprecipitation (RIP) assays, dual-luciferase reporter assays, and rescue experiments were performed to explore the relationships between circRUNX1, miR-449b-5p, Forkhead box protein P3 (FOXP3), and insulin-like growth factor 2 mRNA-binding protein 2 (IGF2BP2).

**Results:**

CircRUNX1 was found to be significantly up-regulated in ESCC tissues and associated with TNM stage and differentiation grade. Functionally, circRUNX1 promoted ESCC cell proliferation and metastasis in vitro and in vivo. CircRUNX1 enhanced FOXP3 expression by competitively sponging miR-449b-5p. Notably, both miR-449b-5p mimics and FOXP3 knockdown restored the effects of circRUNX1 overexpression on cell proliferation and metastasis. Furthermore, IGF2BP2 binding to circRUNX1 prevented its degradation.

**Conclusions:**

IGF2BP2 mediated circRUNX1 functions as an oncogenic factor to facilitate ESCC progression through the miR-449b-5p/FOXP3 axis, implying that circRUNX1 has the potential to be a promising diagnostic marker and therapeutic target for ESCC patients.

**Supplementary Information:**

The online version contains supplementary material available at 10.1186/s13046-022-02550-8.

## Background

Esophageal cancer is the world's seventh most common malignancy in cancer incidence and sixth in cancer mortality, with esophageal squamous cell carcinoma (ESCC) accounting for approximately 90% of cases [1, 2]. ESCC patients are often diagnosed at an advanced stage with lymph node metastasis, resulting in a high mortality rate [3, 4]. Overall, the five-year survival rate for patients with advanced esophageal cancer is frequently less than 20%, and in some countries, it is as low as 5% [5]. Systemic chemotherapy and radiotherapy are the primary treatments for advanced esophageal cancer, but their efficacy has reached a bottleneck due to unknown genetic mechanisms [6]. Therefore, it is critical to elucidate the molecular basis of tumorigenesis and investigate new diagnostic markers and potential therapeutic targets for ESCC.

Circular RNAs (circRNAs), once thought to be the by-product of splicing errors, are single-stranded covalently closed RNA molecules formed by back-splicing of pre-mRNAs [7]. Circular RNAs are more resistant to Ribonuclease R (RNase R) than general linear RNAs due to their unique loop structure lacking 5' G-caps and 3' A-tails, resulting in longer half-lives [8]. Emerging studies have shown that circRNAs play a pivotal role in regulating gene expression at both the transcriptional and post-transcriptional levels [9]. CircRNAs can serve as microRNA (miRNA) sponges, transcription factors, protein scaffolds, and translation templates in some cases [10]. It is worth mentioning that aberrantly expressed circRNAs in multiple tumors are often associated with differentiation grade, TNM stage, and lymph node metastasis (LNM). In addition, they also function as miRNA sponges and are involved in tumor proliferation, migration, apoptosis, and angiogenesis [11–13]. These results suggest that circRNAs have the potential to be used as both diagnostic markers and therapeutic targets for a variety of cancers. However, the functional roles and detailed mechanisms of circRNAs in ESCC remain elusive.

CircRUNX1 is widely reported as a microRNA sponge and plays an important regulatory role in tumorigenesis and metastasis in several tumors. In papillary thyroid cancer (PTC), circRUNX1 promotes PTC progression and metastasis by sponging miR-296-3p and regulating DDHD domain containing 2 (DDHD2) expression [14]. Yu et al. also reported the tumor-promoting effect of circRUNX1/miR-485-5p/Solute carrier family 38 member 1 (SLC38A1) axis [15]. According to previous research, miR-449b-5p acts as a tumor suppressor that is sponged by non-coding RNAs such as linc00667, linc00475, and lncRNA HOTAIR. Aberrant underexpression of miR-449b-5p in various tumors is associated with decreased cell proliferation, migration, and invasion [16–18]. Currently, there is no available research on the biological functions and molecular mechanisms of circRUNX1 and miR-449b-5p in ESCC.

In this study, we constructed circRNA expression profiles in ESCC tissues revealing that circRUNX1 (also named hsa_circ_0002360) was aberrantly expressed in ESCC tissues. Notably, high level of circRUNX1 was correlated with the differentiation grade and TNM stage of ESCC patients. A series of functional investigations showed that circRUNX1 markedly induced the proliferation, migration, and invasion of ESCC cells both in vivo and in vitro. Mechanistically, by functioning as a molecular sponge of miR-449b-5p, circRUNX1 promoted Forkhead box protein P3 (FOXP3) expression at the mRNA and protein levels, consequently resulting in malignant biological behaviors of ESCC cells. In addition, the elevated level of circRUNX1 in ESCC tissues and cells was attributed to its increased stability induced by m6A reader protein Insulin-like growth factor 2 mRNA-binding protein 2 (IGF2BP2). Taken together, our study put forward a promising clinical indicator and therapeutic target for the pathogenesis and treatment of ESCC.

## Methods

### Clinical specimens

Fifty-four pairs of ESCC tissues and paracancerous tissues were collected from patients without preoperative chemotherapy or radiotherapy from the First Affiliated Hospital of Zhengzhou University between 2019 and 2021. The tissues were immediately collected after surgery and stored at -80 °C until use. Written informed consents were obtained from each patient in the cohort.

### Cell culture

Human normal esophageal epithelial cell line HEEC and human ESCC cell lines (KYSE450, EC109, TE1, KYSE70, KYSE150) and HEK-293T cells were purchased from the Shanghai Institute of life science cell bank center (Shanghai, China). All cells were cultured in 1640 medium (Hyclone, USA) with 10% fetal bovine serum (FBS, VivaCell, China) and 1% penicillin–streptomycin (Solarbio, China). Cell plates were incubated at 37 °C and 5% CO_2_ under saturated humidity.

### Cell transfection

Small interfering RNAs targeting circRUNX1 (si-circRUNX1#1, si-circRUNX1#2, and si-circRUNX1#3) and their relative control (si-NC), miR-449b-5p mimic, miR-449b-5p inhibitor, and miR-NC, FOXP3 small interfering RNA (si-FOXP3) and corresponding control (si-NC) were all purchased from RiboBio (Guangzhou, China). All transfections were finished by using Lipofectamine 3000 (Invitrogen, USA). The full-length circRUNX1 was subcloned into the lentivirus vector (Lv‐circRUNX1) and purchased from GeneChem (Shanghai, China). Stably transfected cell lines were filtered by puromycin (2 μg/mL) for two weeks. RT-PCR was performed to detect the transfection efficiency after 48 h. The sequences of siRNA, shRNA, microRNA mimics, and inhibitors were listed in Table S1.

### Total RNA and genomic DNA extraction

Total RNA was separated from ESCC tissues and cell lines using RNAiso Plus (Takara, Japan) as per the manufacturer’s instructions. Genomic DNA (gDNA) was extracted from ESCC cell lines using the Blood/Cell/Tissue Genomic DNA Extraction Kit (TIANGEN, Beijing, China). The integrity and purity of extracted total RNA and gDNA were detected by NanoDrop One (Thermo Fisher Scientific, Waltham, USA). The DNA and RNA products were immediately stored at -80 °C until future use.

### Quantitative real-time polymerase chain reaction (qRT-PCR)

PrimeScript™ RT reagent Kit (Takara, Japan) was used in reverse transcription of total RNA. After removing the genomic DNA at 42 °C for 2 min by gDNA Eraser, total RNA from ESCC tissues and cells was reverse transcribed into cDNA using a RevertAid H Minus First Strand cDNA Synthesis Kit (Thermo Fisher Scientific, Waltham, USA) through the following procedure: 37 °C for 15 min and 85 °C for 5 s. The product was immediately stored at—20 °C until use. The qRT-PCR was conducted on a QuantStudio 5 Real-Time PCR System (Applied Biosystems, Foster City, USA). The procedure is listed as follows: 95 °C for 30 s, 40 cycles of 95 °C for 5 s, and 60 °C for 1 min. The results were calculated by using the relative quantification 2^−ΔΔCT^ method normalized by GAPDH. The primers for qRT-PCR were presented in Table S2.

### Isolation of the cytoplasmic and nuclear fractions

The nuclear and cytoplasmic fractions of RNA were extracted using Cytoplasmic and Nuclear RNA Purification Kit (Norgen Biotek, Japan) according to the manufacturer’s instructions. The extracted RNAs were then used to examine the expression of circRUNX1.

### RNase R treatment

Total RNA (2 μg) extracted from KYSE150 cells was digested with 3 U/μg RNase R (Epicentre, USA) for 15 min at 37℃. The abundance of circRUNX1 and RUNX1 was detected by qRT-PCR.

### Actinomycin D assay

KYSE150 and TE1 cells were treated with 5 μg/mL actinomycin D (Sigma Aldrich, St. Louis, MO, USA) at 0 h, 4 h, 8 h, 12 h, 24 h, respectively. Then cells were harvested to extract total RNA. The stability of circRUNX1 and RUNX1 mRNA was analyzed using qRT-PCR.

### Fluorescencein situhybridization (FISH) assays

Cy3-labeled specific probes to circRUNX1 and positive control probes (RiboBio, Guangzhou, China) were designed and synthesized to detect the location of circRUNX1 in TE1 cells. In brief, after prehybridization for 30 min at 37 ℃, cell crawling sheets were hybridized with 2.5 μL specific Cy3-labelled circRUNX1 probes and positive control probes (20 μM) at 37 °C overnight, and dyed with DAPI. Slides were photographed with confocal laser scanning microscopy (Zeiss, Jena, Germany).

### Western blotting

All cells were lysed with RIPA lysis buffer (Solarbio, China) and protease inhibitors (Beyotime, China). BCA Protein Assay Kit (Biomed, China) was used to adjust proteins to equal concentration. Protein lysates were separated in sodium dodecyl sulfate–polyacrylamide gel electrophoresis (SDS-PAGE) and then transferred to PVDF membranes (Millipore, USA). Subsequently, the membranes were blocked with 5% non-fat milk at room temperature for 2 h and incubated at 4 °C overnight with the primary antibodies against FOXP3 (22228–1-AP, 1:1000, Proteintech, China) or GAPDH (60004–1-Ig, 1:1000, Proteintech, China). ECL chemiluminescent reagent (UE, China) was used for band visualization.

### Immunohistochemistry (IHC) and Hematoxylin and eosin (H&E) staining

The paraffin sections were baked, dewaxed in xylene, and then soaked in gradient ethanol for hydration. After being washed with PBS, samples were heated in boiling citrate buffer (pH 6.0) for 15 min for antigen retrieval. Then sections were incubated at room temperature with 3% H_2_O_2_ for 10 min and blocked with 5% normal goat serum at room temperature for 30 min, then incubated with the primary antibodies anti-IGF2BP2 (11601–1-AP, 1:200, Proteintech, China), anti-FOXP3 (22228–1-AP, 1:200, Proteintech, China), anti-Ki67 (ab15580, 1:200, Abcam, USA) at 4 ℃ overnight. After 2–3 rinses in PBS, the sections were incubated with biotinylated anti-IgG secondary antibody and horseradish peroxidase-labeled streptavidin was added to the slides and incubated for 15 min. The final immunoreactivity score of IGF2BP2 was calculated as previously described [19]. For H&E staining, the nucleus and cytoplasm were stained with hematoxylin and eosin, respectively. General histological examination was performed by standard procedures.

### RNA immunoprecipitation (RIP)

Transfected cells were washed twice in pre-cooled PBS, and then lysed in Polysome lysis buffer. Next, the cell lysates were incubated with Pierce protein A/G magnetic beads (Thermo Scientific, USA) conjugated with anti-IGF2BP2 (11601–1-AP, Proteintech, China) or Ago2 (ab186733, Abcam, USA) in rotator at 4 ℃ overnight. After separation and purification, the immunoprecipitated RNAs were further analyzed by qRT‐PCR to measure the expression of circRUNX1 or miR-449b-5p.

### Luciferase reporter assay

CircRUNX1 or FOXP3 fragments with mutant (Mut) or wild-type (WT) miR-449b-5p binding sites were subcloned into the Renilla gene downstream of the psiCHECK2 dual-luciferase reporter vector (Hanbio, China) resulting in the following constructs: circRUNX1-WT, circRUNX1-Mut, FOXP3 3' UTR-WT and FOXP3 3' UTR-Mut. The plasmids were co-transfected into 293 T cells with miR-449b-5p mimics or NC mimics. After 48 h, the Dual-Luciferase Assay Kit (Beyotime, China) was used to measure relative luciferase activity.

### Colony formation, Cell Counting Kit-8 (CCK-8) and EdU assays

For colony formation assays, KYSE150 or TE1 cells were seeded into 6-well plates at 1200 cells per well and incubated at 37 °C for 2 weeks. As for CCK-8 assays, transfected cells were seeded in 96-well plates. 10 μL of cell Counting Kit-8 (Servicebio, Wuhan, China) were added to 96-well at 37 °C for 2 h, then the absorbance was measured at 450 nm by a microplate reader (SpectraMax i3x, Molecular Devices, Shanghai, China). Cell-Light EdU Apollo567 In Vitro Kit (RiboBio, Guangzhou, China) was used in EdU assays and performed as previously described [20]. In brief, KESE150 and TE1 cells were cultured with EdU solution at 37 °C for 4 h. After fixation and penetration, cells were treated with Apollo dyes and DAPI for EdU staining and nuclear staining. Finally, cell images were captured by Inverted Fluorescence Microscope (Olympus, Tokyo, Japan).

### Transwell and wound-healing assays

For transwell assays, ESCC cells were seeded into the upper chambers with or without Matrigel (Corning, NewYork, USA), then the chambers were put into 1640 medium with 20% fetal bovine serum for 48 h. As for wound-healing assays, 2 × 10^4^ cells were seeded into 12-well plates for 12 h. Then 10 μL pipette tip was used to make a gap, and serum-free medium was added into 12-well plates to culture cells for another 24 h.

### Animal experiments

Four to five-week-old BALB/c nude mice (Shanghai SLAC Laboratory Animal Co. Ltd.) were used to construct in vivo tumor models. 5 × 10^6^ KYSE150 cells were suspended in 100 μL of PBS and injected subcutaneously into the mice. The size of tumors was measured every 5 days and the volume of tumors was calculated as follows: 0.5 × length × width^2^. After 30 days, the mice were sacrificed and the tumors were isolated. Separated tumors were immediately fixed with 4% paraformaldehyde for subsequent experiments. For in vivo metastasis model, 2 × 10^6^ KYSE150 cells were injected into the tail veins to establish pulmonary metastasis models. These nude mice were monitored weekly and euthanized 60 days later, then pulmonary nodules in each group were counted and fixed.

### Statistical analysis

All experimental data were displayed as mean ± standard deviation (SD). Comparisons between different groups were analyzed using unpaired Student's t-test or one-way variance (ANOVA). The expression of indicated molecules in ESCC tissues and corresponding paracancerous tissues were compared using paired t-test. The association between circRUNX1 and miR-449b-5p, FOXP3, IGF2BP2 was analyzed using Pearson correlation test. *P* < 0.05 was considered statistically significant. All data were analyzed using GraphPad Prism 9.0 software.

## Results

### Expression and characterization of circRUNX1 in ESCC tissues and cell lines

**Fig. 1:**
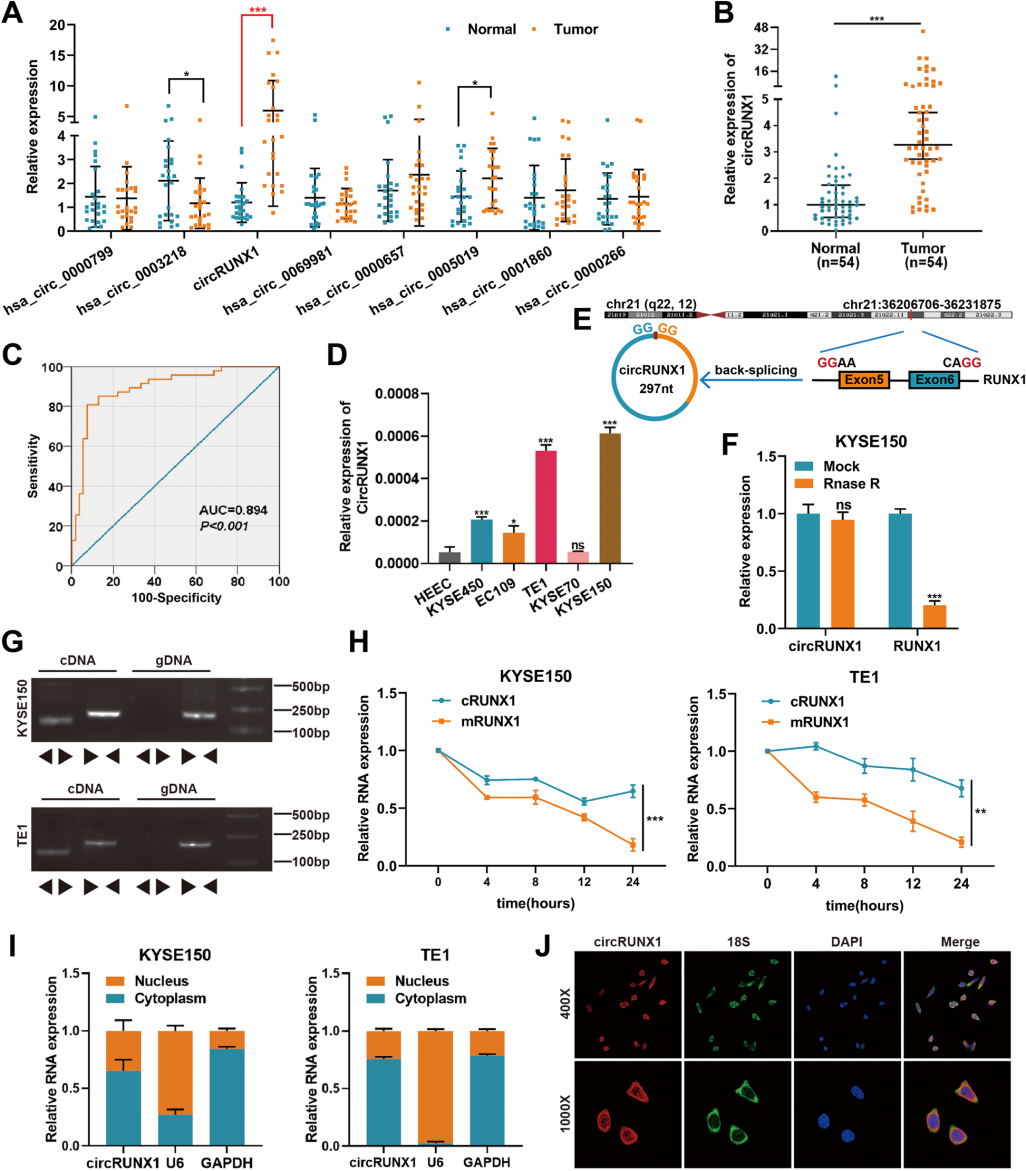
Expression and validation of circRUNX1 in ESCC tissues and cells. **A** Expression of top eight circRNAs in 25 pairs of ESCC tissues was detected by qRT-PCR. **B** qRT-PCR analysis of circRUNX1 expression in 54 pairs of ESCC tissues (25 pairs in panel A and another 29 pairs of ESCC tissues). **C** Diagnosed value of circRUNX1 was displayed in ROC curve. **D** Relative expression of circRUNX1 in a panel of ESCC cell lines and normal esophageal epithelial cells (HEEC). **E** Schematic illustration of circRUNX1 formation via the circularization from exon 5 to exon 6 in the RUNX1 gene located on chromosome 21. **F** The expression of circRUNX1 and RUNX1 mRNAs after treatment with RNase R in KYSE150 cells. **G** Convergent and divergent primers were used to verify closed loop structure of circRUNX1. **H** The expression changes of circRUNX1 (cRUNX1) and RUNX1 (mRUNX1) were detected by qRT-PCR in KYSE150 and TE1 cells treated with actinomycin D at the indicated time points. **I** The abundance of circRUNX1 in the nuclear and cytoplasmic of KESE150 and TE1 cells was measured by qRT-PCR. **J** FISH assays for circRUNX1 in KESE150 and TE1 cells. Nuclei were stained with DAPI and cytoplasm was staind with 18S. (Magnification × 400. Scale bar: 20 μm and Magnification × 1000. Scale bar: 10 μm). The data are presented as the mean ± SD. *P < 0.05, **P < 0.01, ***P < 0.001

To explore circRNAs expression profiles in ESCC, circRNA sequencing was performed on cancer tissues and corresponding paracancerous tissues. Sequencing results revealed a total of 581 differentially expressed circRNAs in ESCC tissues (fold change ≥|2.0| and *P* ≤ 0.05), including 261 up-regulated circRNAs and 320 down-regulated circRNAs. Among the top 8 most high-expressed circRNAs, we found that circRUNX1 (hsa_circ_0002360) was substantially overexpressed in 25 pairs of ESCC tissues (Fig. 1A and Table S3). Then we validated the expression of circRUNX1 in 54 pairs of ESCC tissues and paired normal esophageal tissues by qRT-PCR (Fig. 1B). Taking the median as the boundary, the 54 patients were divided into the high expression group (*n* = 27) and the low expression group (*n* = 27). Analysis of clinicopathological features revealed that high circRUNX1 expression was significantly associated with advanced TNM stage and poor differentiation grade (Table S4 and Fig. S1). Based on the expression of circRUNX1 in ESCC tissues, a Receiver Operating Characteristic (ROC) curve analysis was constructed, and the results revealed an Area Under Curve (AUC) of 0.894 (95% CI: 0.829–0.960, *P* < 0.001), indicating that circRUNX1 could be used to distinguish cancer and paracancerous tissues in ESCC patients (Fig. 1C). Next, the circRUNX1 expression in ESCC cell lines was investigated. As shown in Fig. 1D, circRUNX1 was highly expressed in KYSE150 and TE1 cell lines compared to normal esophageal epithelium cell line, HEEC. As a result, these two cell lines were used for subsequent investigations. As depicted in Fig. 1E, circRUNX1 is derived from exon 5 and exon 6 of Runt-related transcription factor (RUNX1) pre-mRNA by head-to-tail splicing. qRT-PCR revealed that circRUNX1 was more resistant to RNase R digestion than its parental mRNA (Fig. 1F). To verify its ring structure, we designed divergent primers and convergent primers. The agarose gel electrophoresis results showed that circRUNX1 in cDNA could be amplified not only by divergent primers but also by convergent primers. However, no amplification product was found in gDNA amplified with divergent primers (Fig. 1G). After 24 h of actinomycin D treatment, we discovered that the half-life of circRUNX1 was much longer than that of RUNX1 mRNA in KYSE150 and TE1 cell lines (Fig. 1H). To investigate its localization, qRT-PCR was utilized to detect the distribution of circRUNX1 in the nucleus and cytoplasm. The results revealed that a significant portion of circRUNX1 was localized in the cytoplasm (Fig. 1I), and FISH assay further confirmed that the majority of circRUNX1 was located in the cytoplasm of TE1 cells (Fig. 1J). Taken together, our results indicated that increased expression of circRUNX1 correlated with the advanced TNM stage and poor differentiation grade of individuals with ESCC.

### CircRUNX1 induces ESCC cell malignant behaviors in vitro

**Fig. 2:**
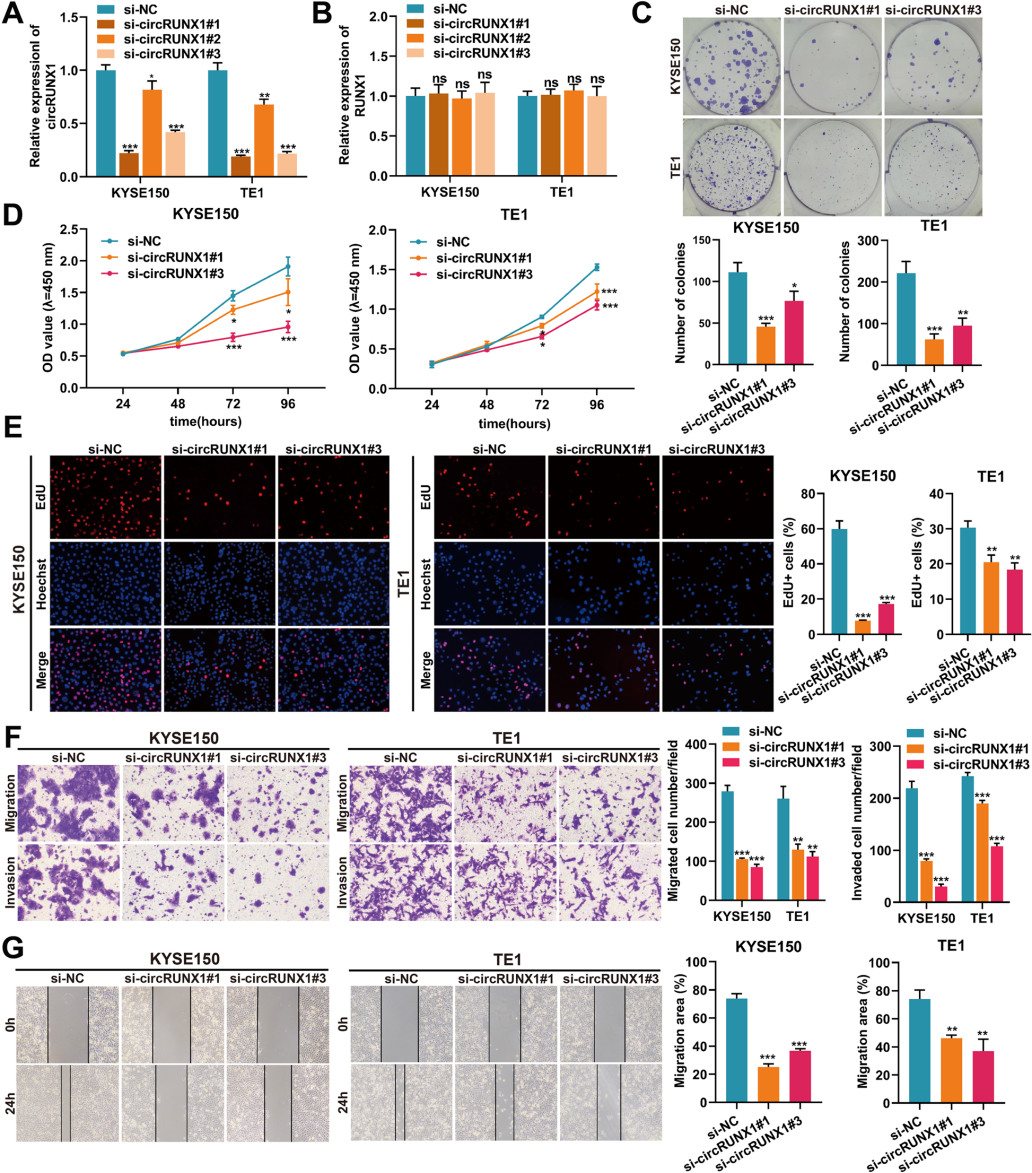
CircRUNX1 knockdown suppresses ESCC progression. **A** and** B** Expression levels of circRUNX1 (**A**) and RUNX1 (**B**) post transfection of three siRNAs targeting circRUNX1. **C** Colony formation assays were performed in ESCC cell lines with circRUNX1 knockdown. **D** The cell viability of KYSE150 and TE1 cells were evaluated by CCK-8 assays. **E** Changes of cell proliferation after knocking down circRUNX1 were detected by EDU assay (Magnification × 100. Scale bar: 100 μm). **F** Migration and invasion abilities of KYSE150 and TE1 cells after circRUNX1 inhibition were evaluated by transwell assays. **G** Migration abilities of KYSE150 and TE1 cells after depletion of circRUNX1 were evaluated by wound healing assay. **P < 0.01, ***P < 0.001

To investigate the biological function of circRUNX1 in ESCC cells, circRUNX1 was depleted using three siRNAs that targeted its back-splicing junction sites, and stably overexpressed circRUNX1 cell lines were established utilizing GV-689 lentivirus vector (Fig. 2A and Fig. S2A). Neither siRNAs nor GV-689-circRUNX1 affected linear RUNX1 expression (Fig. 2B and Fig. S2B). CCK-8 assays revealed that ESCC cell viability was inhibited in KYSE150 and TE1 cells after circRUNX1 depletion but significantly increased after circRUNX1 overexpression (Fig. 2D and Fig. S2C). Moreover, colony formation and EdU assays demonstrated that downregulation of circRUNX1 suppressed cell proliferation and limited the colony formation ability of KYSE150 and TE1, while ectopic expression of circRUNX1 showed the opposite trend (Fig. 2C, E and Fig. S2D, E, H). Additionally, the effects of circRUNX1 on the migratory and invasive abilities of KYSE150 and TE1 cells were further examined using transwell and wound healing assays. As shown in Fig. 2F, G and Fig. S2F, G, circRUNX1 knockdown significantly restricted the migration and invasion of ESCC cells, whereas circRUNX1 overexpression had the opposite effect. Collectively, the above data suggested that circRUNX1 played a carcinogenic role in ESCC cells.

### FOXP3 is a downstream target of circRUNX1 to accelerate ESCC progression

**Fig. 3:**
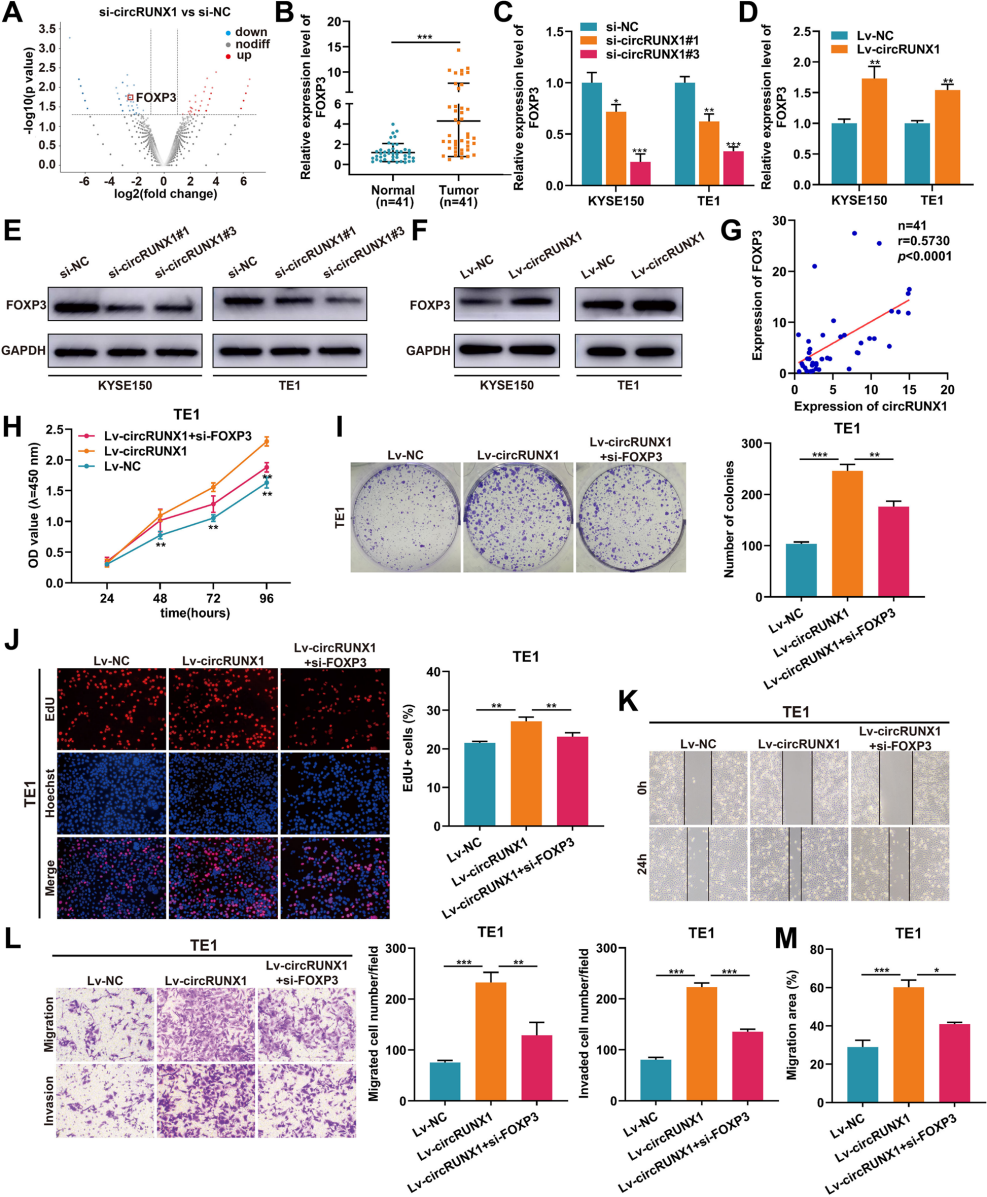
CircRUNX1 promotes cell proliferation and metastasis by upregulating FOXP3 expression. **A** Volcano plot showing differentially expressed genes in TE1 cells transfected with si-circRUNX1 and si-NC.** B** Differential expression of FOXP3 in 41 pairs of ESCC tissues and matched esophageal normal tissues. **C** and** D** Relative mRNA levels of FOXP3 were detected in ESCC cells with circRUNX1 knockdown or overexpression using qRT-PCR.** E** and** F** Relative protein levels of FOXP3 after inhibition or ectopic expression of circRUNX1 were determined using western blotting. **G** The correlation between circRUNX1 and FOXP3 was detected in 41 clinical ESCC samples. **H-J** CCK-8 (**H**), colony formation (**I**) and EdU assays (**J**) were performed to examine the proliferative effects of circRUNX1 and FOXP3 on TE1 cells. **K-M** Wound healing and transwell assays were adopted to evaluate the migration and invasion capabilities of TE1 cells after overexpression of circRUNX1 or disruption of  FOXP3. *P < 0.05, **P < 0.01, ***P < 0.001

To depict the potential downstream regulatory mechanisms of circRUNX1 in ESCC cells, RNA-seq was utilized to determine the differentially expressed transcriptomes in TE1 cells with circRUNX1 knocking down and control cells, which identified 90 downregulated genes and 76 upregulated genes (Fig. 3A). Gene Ontology (GO) functional annotation and KEGG enrichment analysis assessed the differentially expressed genes involved in the regulation of cancers (Fig. S3A-C). Of the 90 downregulated genes, 44 genes have not been annotated and seven genes were long non-coding RNA (lncRNA) (Fig. S3D). To explore the potential target genes of circRUNX1 among the remaining 39 genes, GEPIA (http://gepia.cancer-pku.cn/) was used to predict the expression of these genes in ESCC patients. Results showed that only three genes (IFI44, CTSE and FOXP3) were upregulated in ESCC (Fig. S3E). Next, we detected the changes in the expression levels of these three genes in circRUNX1 knocked down KYSE150 and TE1 cells. As shown in Fig. 3C and Fig. S3F, FOXP3 was significantly downregulated after interfering with circRUNX1 and was therefore selected for further investigation. RT-PCR assay showed that FOXP3 was highly expressed in 41 pairs of ESCC tissues compared with corresponding paracancerous tissues in our cohort (Fig. 3B). The analysis result of Starbase (https://starbase.sysu.edu.cn) further validated this finding (Fig. S3G). Moreover, GEPIA website demonstrated that FOXP3 expression was related to the poor prognosis of ESCC patients (Fig. S3H). Subsequently, qRT-PCR and western blot analysis revealed that circRUNX1 could positively regulate the expression of FOXP3 in ESCC cells (Fig. 3C-F). In addition, FOXP3 mRNA level in ESCC tissues was moderately related to circRUNX1 transcripts (*r* = 0.5730, *P* < 0.0001) (Fig. 3G). Considering that the role of FOXP3 in ESCC cells remains largely unknown, we knocked down FOXP3 in KYSE150 and TE1 cells (Fig. S4A and B). CCK-8 assays revealed that FOXP3 depletion significantly reduced cell proliferation in KYSE150 and TE1 cells (Fig. S4C). Moreover, transwell and wound healing assays confirmed that FOXP3 knockdown inhibited KYSE150 and TE1 cell migration and invasion (Fig. S4D and E). The above data suggested that FOXP3 acted as a tumor promoter in ESCC cells. Given that circRUNX1 could increase FOXP3 expression, we wondered whether FOXP3 was involved in circRUNX1-mediated ESCC progression. To this end, FOXP3 expression was inhibited in KYSE150 and TE1 cells stably expressing circRUNX1. Overexpression of circRUNX1 increased the proliferative ability of KYSE150 and TE1 cells in CCK-8, colony formation, and EdU assays, while silencing FOXP3 reversed these phenotypes (Fig. 3H-J and Fig. S5A-C). Meanwhile, FOXP3 inhibition reduced circRUNX1-induced ESCC cell mobility, as demonstrated by transwell and wound healing experiments (Fig. 3K-M and Fig. S5D-F). Together, these findings showed that FOXP3 functioned as a downstream target of circRUNX1 in ESCC cells.

### CircRUNX1 acts as a molecular sponge of miR-449b-5p

**Fig. 4:**
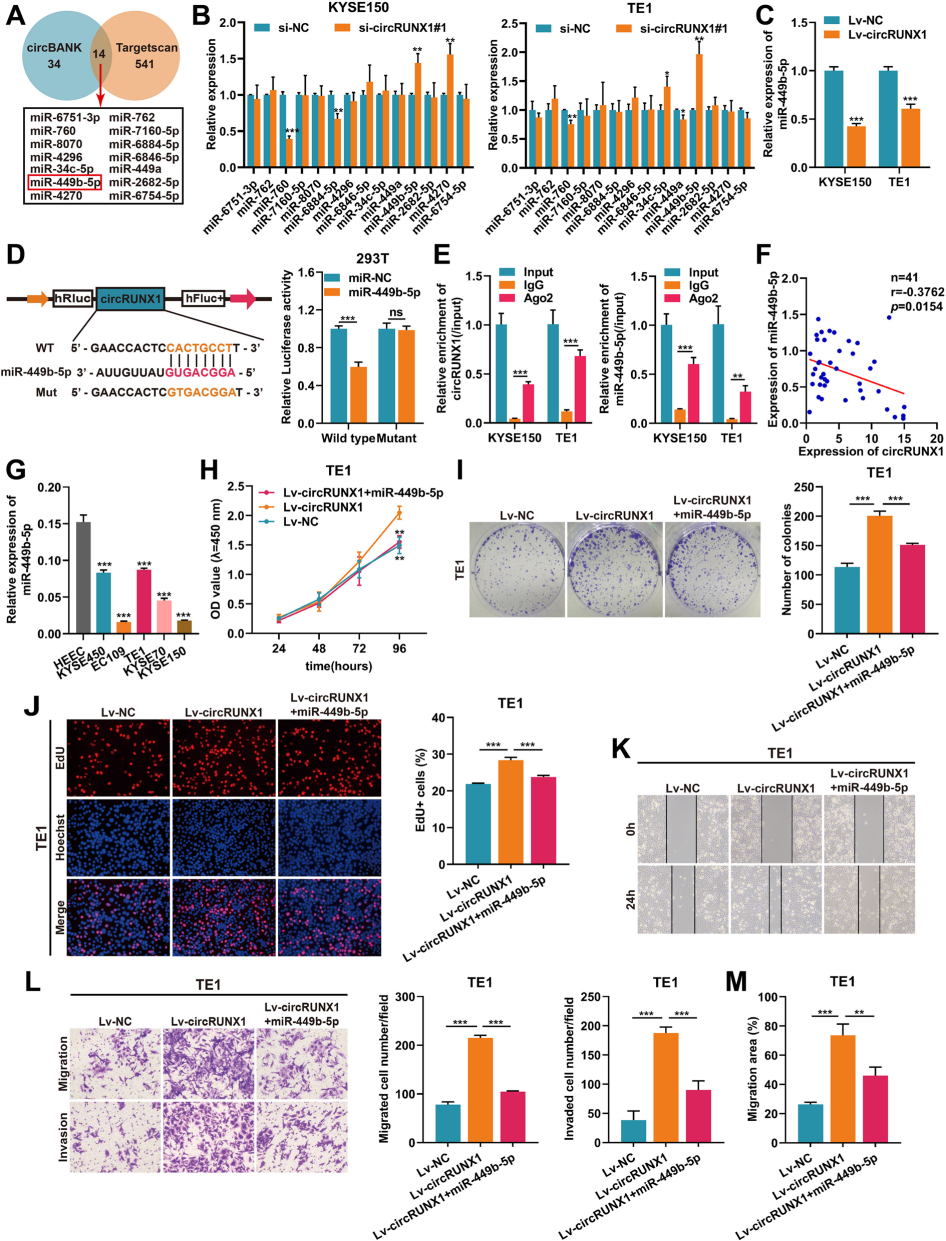
CircRUNX1 serves as miR-449b-5p sponge to promote ESCC progression. **A** Venn diagram showing the candidate miRNAs predicted to be the binding targets of circRUNX1 and FOXP3 by circBank (http://www.circbank.cn) and Targetscan (http://www.targetscan.org). **B** Relative expression of 14 candidate miRNAs after knocking down circRUNX1. **C** Relative expression of miR-449b-5p in KYSE150 and TE1 cells stably expressing circRUNX1. **D** The luciferase activity was determined in 293 T cells co-transfected with miR-449b-5p mimics/miR-NC and circRUNX1-WT/circRUNX1-MUT. **E** Anti-Ago2 RIP assays were performed in KYSE150 and TE1 cells to detect the enrichment ability of Ago2 to circRUNX1 and miR-449b-5p. **F** The correlation between miR-449b-5p and circRUNX1 expression in 41 primary ESCC tissues. **G** Relative expression of miR-449b-5p in a panel of ESCC cell lines and normal esophageal epithelial cells (HEEC). **H-J** CCK-8 (**H**), colony formation (**I**) and EdU assays (**J**) were performed to measure the proliferative abilities of TE1 cells transfected with circRUNX1 or circRUNX1 plus miR-449b-5p mimics. **K-M** Wound healing and transwell assays were utilized to evaluate the migration and invasion capacities of TE1 cells transfected with indicated vectors. Data are represented as the mean ± SD from three independent experiments. *P < 0.05, **P < 0.01, ***P < 0.001

One of the most well-known circRNA mechanisms is its role as a competing endogenous RNA (ceRNA) for miRNA binding [21]. Since circRUNX1 was found primarily in the cytoplasm, we hypothesized that it might regulate FOXP3 levels by sponging miRNAs. Two bioinformatics databases (circBank and Targetscan) were utilized to predict potential targeting miRNAs bound by circRUNX1 and FOXP3, and 14 miRNAs were identified in the overlapping list (Fig. 4A). We further examined the expression of these miRNAs in circRUNX1 knockdown ESCC cells. After depleting circRUNX1, only one miRNA, miR-449b-5p, was up-regulated in KYSE150 and TE1 cells, according to qRT-PCR results (Fig. 4B). Correspondingly, ESCC cells stably transfected with circRUNX1 showed a lower expression of miR-449b-5p (Fig. 4C). The dual-luciferase reporter gene assays further showed that miR-449b-5p mimics markedly reduced the luciferase activities of wild-type circRUNX1 reporter but not mutant circRUNX1 in HEK-293 T cells (Fig. 4D). RIP assays confirmed that both circRUNX1 and miR-449b-5p could directly bind to Ago2 (Fig. 4E). Moreover, we analyzed the correlation between circRUNX1 and miR-449b-5p in ESCC tissues, and the result indicated that miR-449b-5p levels were negatively correlated with the expression of circRUNX1 (Fig. 4F). Consistently, miR-449b-5p expression was also significantly decreased in ESCC cell lines (KYSE450, EC109, TE1, KYSE70, KYSE150) compared with normal esophageal epithelium cell line (HEEC) (Fig. 4G). Next, rescue experiments were conducted to determine whether circRUNX1 promoted ESCC progression by competitively interacting with miR-449b-5p. As shown in Fig. 4H-J and Fig. S6A-C, after stably transfecting circRUNX1 in KYSE150 and TE1 cells, cell proliferation and clonogenic abilities were dramatically enhanced. However, these effects could be attenuated by restoring miR-449b-5p expression. Additionally, transwell and wound healing assays showed that the promoting effects of circRUNX1 on cell migration and invasion were abolished after transfecting miR-449b-5p in KYSE150 and TE1 cells (Fig. 4K-M and Fig. S6D-F). Thus, these findings suggested that circRUNX1 acted as a miR-449b-5p sponge to promote ESCC proliferation and metastasis.

### CircRUNX1 promotes ESCC progression through the circRUNX1/miR-449b-5p/FOXP3 axis

**Fig. 5:**
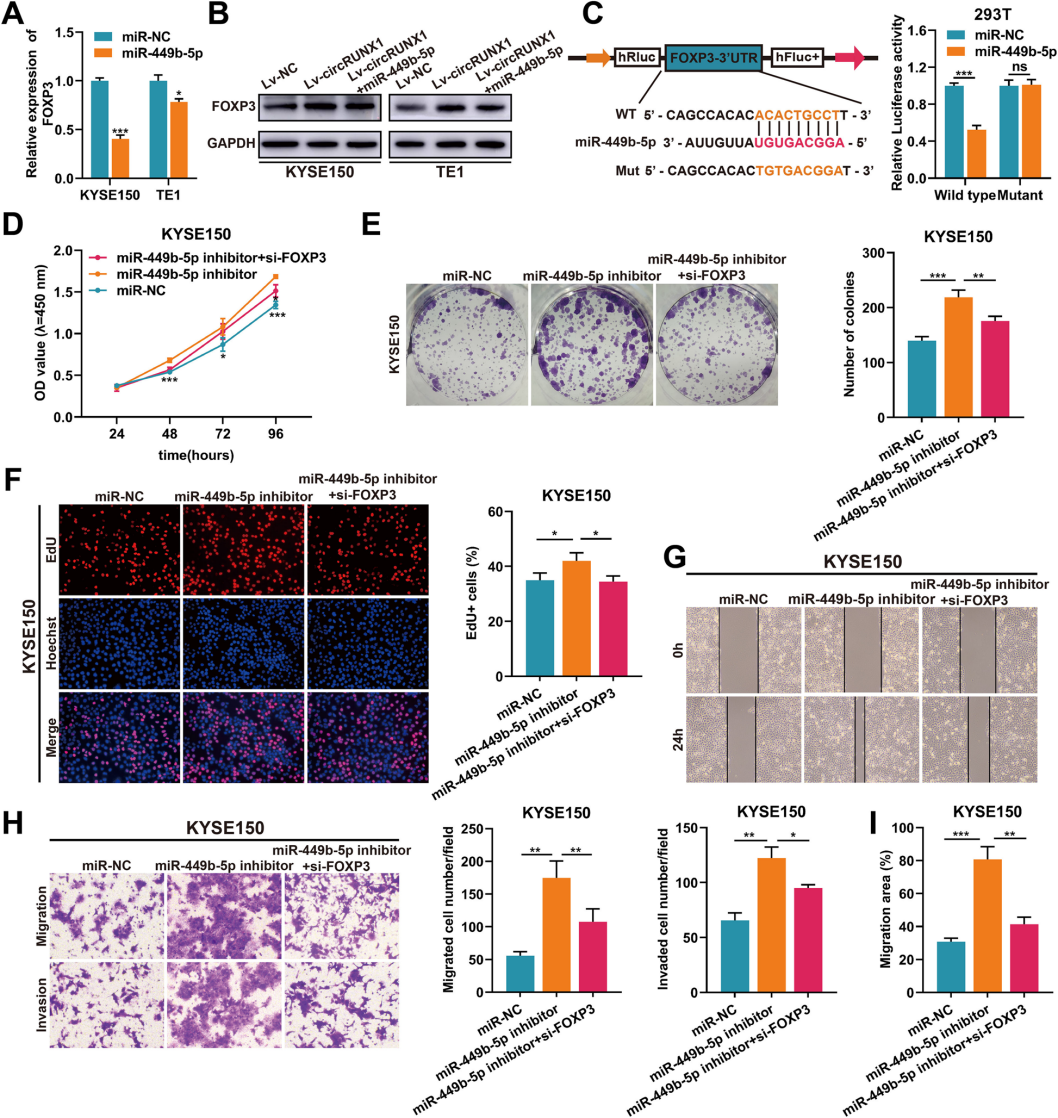
CircRUNX1/miR-449b-5p/FOXP3 axis facilitates ESCC proliferation and metastasis. **A** Relative expression of FOXP3 in KYSE150 cells and TE1 cells transfected with miR-449b-5p mimics or miR-NC. **B** Western blot analysis of FOXP3 level in ESCC cells after transfecting miR-449b-5p mimics or miR-NC. **C** The luciferase activity was determined in 293 T cells co-transfected with miR-449b-5p mimics/miR-NC and FOXP3-WT/FOXP3-MUT. **D-F** CCK-8 (**D**), colony formation (**E**) and EdU assays (**F**) were performed to measure the proliferative effects of miR-449b-5p and FOXP3 on KYSE150 cells. **G-I** The migratory or invasion properties of the miR-449b-5p inhibition with or without FOXP3 depletion in KYSE150 cells and control cells were analyzed by wound healing and transwell experiments. Data are represented as the mean ± SD from three independent experiments. *P < 0.05, **P < 0.01, ***P < 0.001

Next, we transfected miR-449b-5p mimics in KYSE150 and TE1 cells to examine the change in FOXP3 expression. qRT-PCR revealed that the mRNA levels of FOXP3 were downregulated simultaneously after transfecting miR-449b-5p mimics (Fig. 5A). In addition, miR-449b-5p antagonized the upregulation of FOXP3 protein levels induced by circRUNX1 (Fig. 5B). To test whether miR-449b-5p could bind to the 3’UTR of FOXP3, luciferase reporters with wild-type FOXP3 (FOXP3-WT) and mutant FOXP3 (FOXP3-Mut) were constructed and transfected into HEK-293 T cells. The results of the dual-luciferase assay showed that miR-449b-5p mimics could reduce the luciferase activity of FOXP3-WT reporter rather than FOXP3-Mut reporter (Fig. 5C). Based on the above results, we surmised that circRUNX1 served its biological function via circRUNX1/miR-449b-5p/FOXP3 axis. To test this conjecture, KYSE150 and TE1 cells were treated with miR-NC, miR-449b-5p inhibitor, or miR-449b-5p inhibitor plus si-FOXP3. CCK-8, colony formation, and EdU assays showed that knockdown of FOXP3 resisted the growth-promoting effects caused by miR-449b-5p inhibitor (Fig. 5D-F and Fig. S7A-C). In addition, miR-449b-5p inhibitor led to an obvious enhancement in the migration and invasion of KYSE150 and TE1 cells, while FOXP3 downregulation partially mitigated this effect, as demonstrated by wound healing and transwell assays (Fig. 5G-I and Fig. S7D-F). Collectively, the above findings showed that circRUNX1 acted as a miR-449b-5p sponge to promote ESCC proliferation and metastasis by up-regulating FOXP3 expression.

### Elevated IGF2BP2 regulates the stability of circRUNX1 in ESCC

**Fig. 6:**
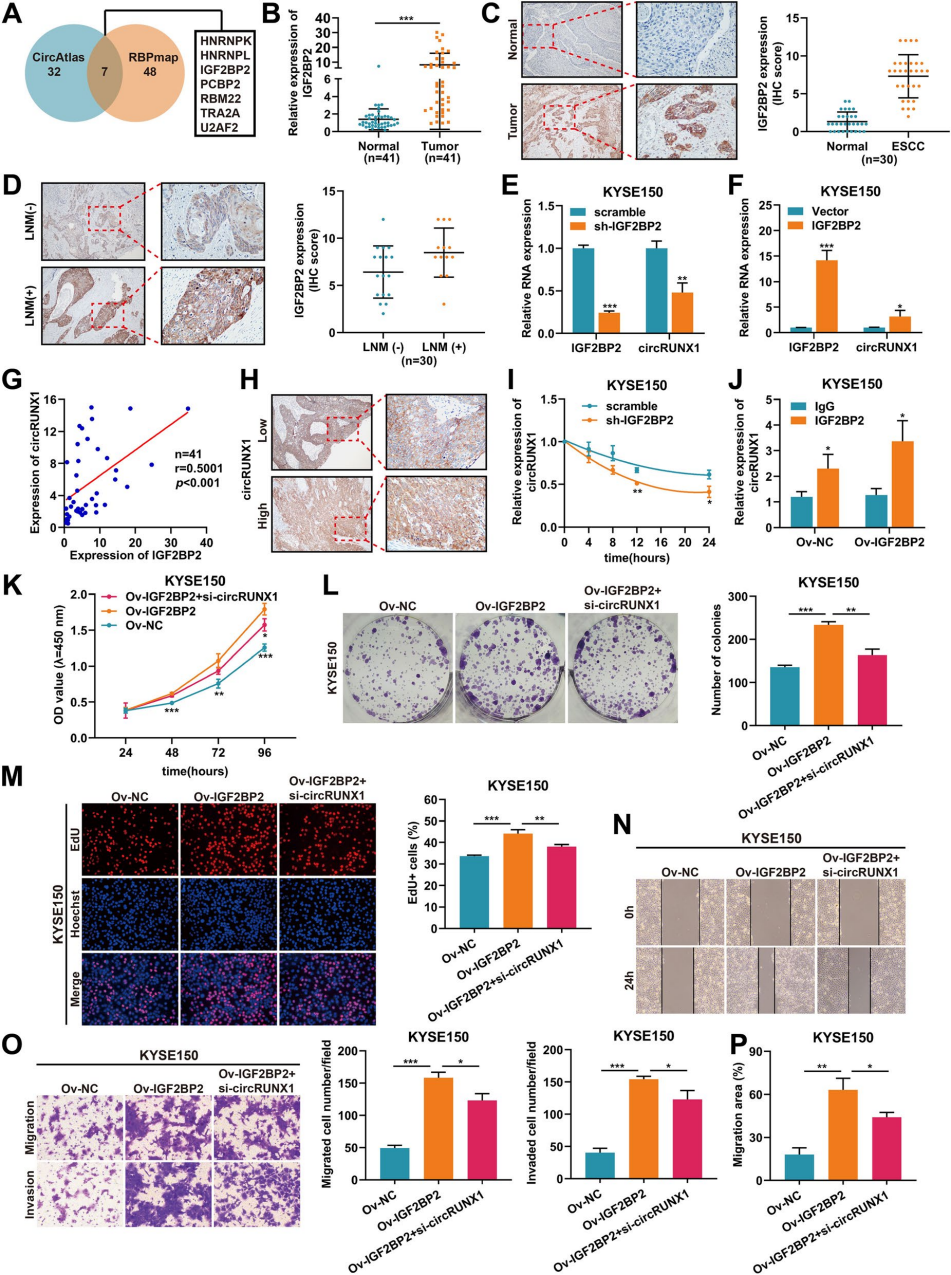
IGF2BP2 silencing retards ESCC progression via accelerating circRUNX1 degradation. **A** Venn diagram showing the candidate RNA binding proteins predicted to combine with circRUNX1by circAtlas (http://circatlas.biols.ac.cn) and RBPmap (http://rbpmap.technion.ac.il).** B** IGF2BP2 mRNA levels were detected by qRT-PCR in 41 pairs of ESCC tissues and their corresponding normal tissues. **C** Expression and scoring of IGF2BP2 protein were detected by IHC in 30 pairs of ESCC tissues. **D** IGF2BP2 immunostaining scores were detected by IHC in ESCC tissues with lymph node metastasis (*n* = 13) and non-metastasis (*n* = 17). **E** and** F** Changes of circRUNX1 and IGF2BP2 levels were determined in KYSE150 cells upon silencing or overexpression of IGF2BP2. **G** The correction between circRUNX1 and IGF2BP2 were examined in 41 ESCC tissues. **H** IHC results showing that higher circRUNX1 expression was related to higher abundance of IGF2BP2 protein in ESCC tissues. **I** qRT-PCR analysis showing circRUNX1 expression in KYSE150 cells treated with actinomycin D for specific time points. **J** RIP assay using anti-IGF2BP2 antibody and IgG antibody was executed in KYSE150 cells with or without overexpressing IGF2BP2. **K-M** CCK-8 (**K**), colony formation (**L**) and EdU assays (**M**) were conducted to investigate the proliferative abilities of KYSE150 cells with IGF2BP2 overexpression or circRUNX1 inhibition. **N-P** Wound healing and transwell assays showing the migration and invasion capabilities of KYSE150 cells with IGF2BP2 overexpression or circRUNX1 inhibition. *P < 0.05, **P < 0.01, ***P < 0.001

To understand why circRUNX1 was overexpressed in ESCC, two bioinformatics databases (circAtlas and RBPmap) were searched, and six proteins with the potential to bind to circRUXN1 at its flanking regions were identified (Fig. 6A). Then the expression of these six proteins in ESCC patients was predicted by GEPIA. Interestingly, only the expression of IGF2BP2 differed between ESCC and normal esophageal tissues, as shown in Fig. S8. The results were in line with the ESCC samples that were analyzed using RT-PCR and IHC staining in our cohort (Fig. 6B and C). Besides, we noticed that IGF2BP2 overexpression in ESCC tissues was significantly associated with lymph node metastasis (Fig. 6D and Table S5). Next, we designed the overexpression vector and specific short hairpin RNA (shRNA) targeting IGF2BP2 and transfected them into ESCC cells. As expected, qRT-PCR results showed that knocking down or overexpressing IGF2BP2 could decline or increase the levels of circRUNX1 (Fig. 6E and F). Next, the positive relationship between circRUNX1 and IGF2BP2 was observed in 41 ESCC tissues (*r* = 0.5001, *P* < 0.001), and IHC staining also validated this result (Fig. 6G and H). To determine whether IGF2BP2 promoted circRUNX1 formation or inhibited circRUNX1 degradation, KYSE150 cells transfected with scramble or sh-IGF2BP2 were treated with actinomycin D for the specific time. The change in circRUNX1 expression levels was then measured upon IGF2BP2 inhibition. Notably, qRT-PCR analysis revealed that silencing IGF2BP2 significantly reduced the half-life of circRUNX1 (Fig. 6I). Subsequently, RIP assays confirmed the direct interaction between IGF2BP2 and circRUNX1 in ESCC cells (Fig. 6J). Next, we performed rescue experiments aiming to verify the biological effects of IGF2BP2 on ESCC progression. The proliferative abilities of KYSE150 and TE1 cells were tested using CCK-8, colony formation, and EdU assays. As shown in Fig. 6K-M and Fig. S9A-C, upregulation of IGF2BP2 increased cell viability, the number of cell colonies, and the percentage of proliferating cells. However, these promoting effects of IGF2BP2 were evidently impaired by circRUNX1 knockdown. Additionally, transwell and wound healing assays revealed that loss of circRUNX1 diminished Ov-IGF2BP2-mediated enhancement of migration and invasion in KYSE150 and TE1 cells (Fig. 6N-P and Fig. S9D-F). Taken together, these findings suggested that IGF2BP2 acted as an upstream target of circRUNX1 and promoted its expression by increasing RNA stability.

### CircRUNX1 promotes proliferation and metastasis in a FOXP3-dependent manner in vivo

**Fig. 7:**
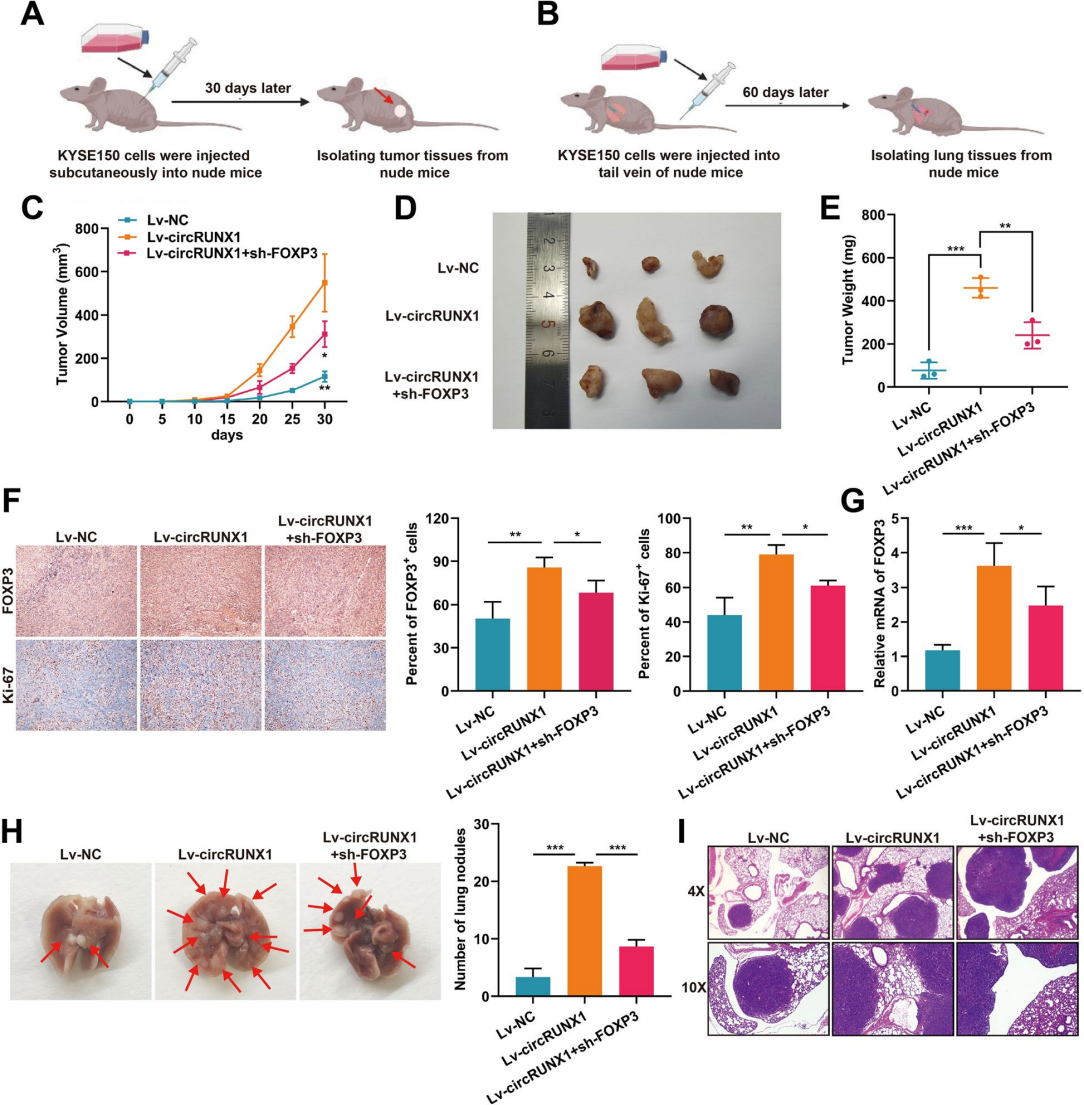
CircRUNX1 targets FOXP3 to accelerate tumorigenesis and metastasis in vivo. **A** and** B** The process of constructing subcutaneous tumor models (**A**) and pulmonary metastasis models (**B**) in nude mice. **C** Tumor volumes of indicated groups were measured every 5 days. **D** Image of subcutaneous tumors from three groups were displayed. **E** The weights of subcutaneous tumors were quantified. **F** Representative IHC staining images and quantification of FOXP3 and Ki-67 positive cells in the indicated xenograft tumors. Magnification × 100. **G** FOXP3 mRNA levels of subcutaneous tumor tissues were detected by qRT-PCR. **H** The images of lung metastases and statistical analysis of metastatic lung foci. **I** Representative images from H&E staining of the lung metastasis nodules. *P < 0.05, **P < 0.01, ***P < 0.001

**Fig. 8:**
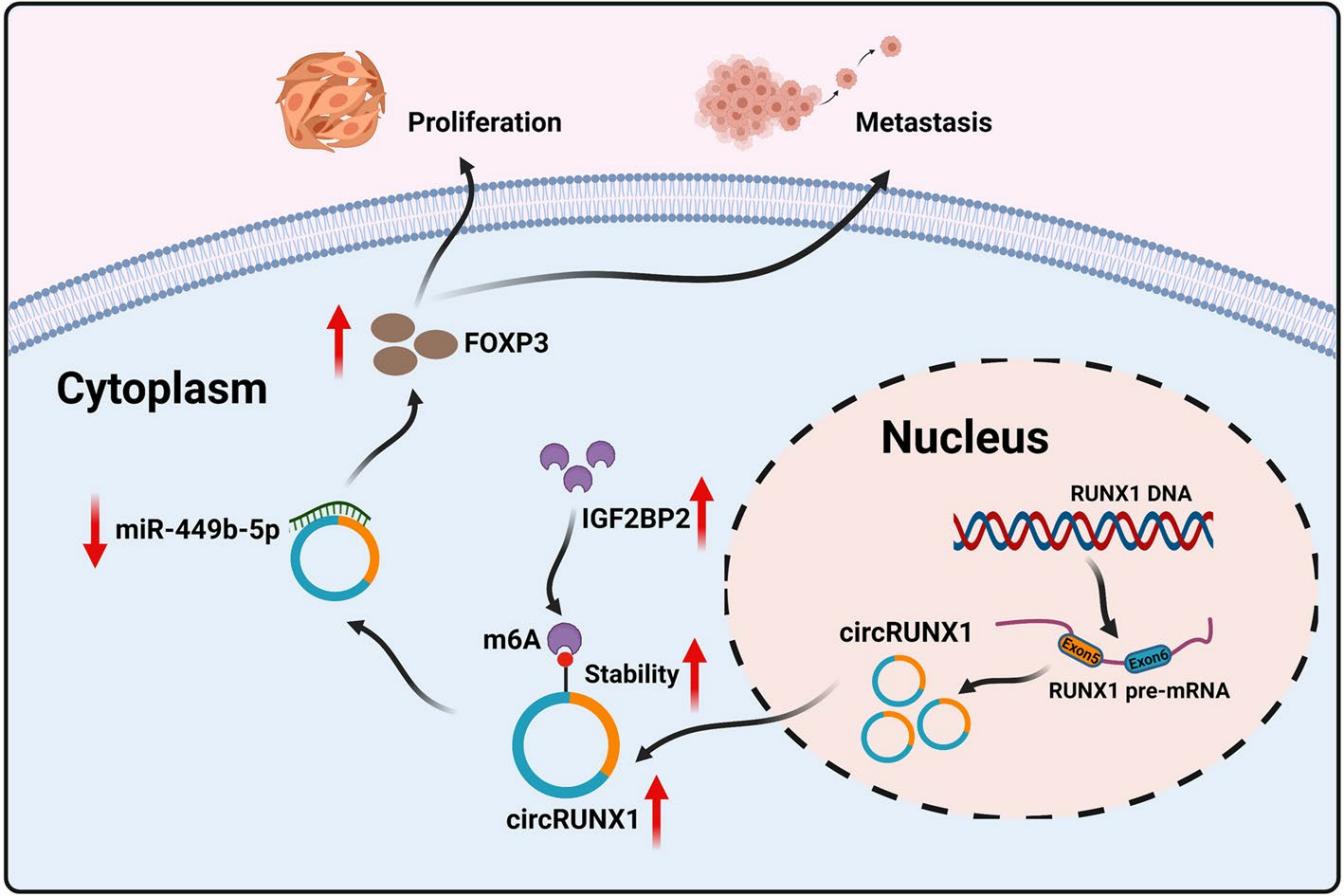
Schematic illustration of the biological role of IGF2BP2/circRUNX1/miR-449b-5p/FOXP3 axis in modulating ESCC malignant progression

Next, we further investigated the specific roles of circRUNX1 and FOXP3 in vivo based on the findings described above. KYSE150 cells stably expressing circRUNX1 or co-transfected with circRUNX1 and FOXP3 shRNA were injected subcutaneously or intravenously into nude mice (Fig. 7A and B). After 30 days of observation, we noticed that circRUNX1 overexpression groups showed significantly faster tumor growth than the other two groups, while inhibition of FOXP3 in circRUNX1-overexpressing cells restricted tumor growth (Fig. 7C and D). This conclusion was also supported by the final tumor weights of the three groups (Fig. 7E). Next, the levels of FOXP3 and Ki-67 from these tumor tissues were detected by IHC staining. As shown in Fig. 7F, circRUNX1 increased FOXP3 and Ki-67 expression, while FOXP3 deletion reduced this effect. RT-PCR analysis of these xenograft tissues also revealed that overexpression of circRUNX1 increased the endogenous level of FOXP3 (Fig. 7G). Pulmonary metastasis models were then established by inoculating KYSE150 cells into the tail-vein of nude mice. Lung tissues from each group were retrieved at the end of the experiment, and H&E staining revealed that overexpression of circRUNX1 induced KYSE150 cells to form more lung metastases in vivo. In contrast, the number of lung foci was reduced after co-transfecting ESCC cells with sh-FOXP3, indicating that circRUNX1 and FOXP3 have pro-metastatic effects (Fig. 7H and I). Taken together, for the first time, the biological function and specific mechanism of circRUNX1 in ESCC progression have been identified (Fig. 8).

## Discussion

So far, only a few circRNAs have been identified as having various functions in ESCC. Peng et al. found that circPDE3B could function as an oncogenic factor by harboring miR-4766-5p to eliminate the inhibitory effect on the target gene laminin α1 (LAMA1) [22]. Another report revealed that circ-Foxo3 inhibited ESCC progression via the miR-23a/phosphatase and tensin homolog (PTEN) axis [23]. In addition, certain circRNAs were also involved in regulating the radiosensitivity of ESCC cells, including hsa_circ_0014879 [24] and circ-0007022 [25]. However, the roles of a large number of circRNAs in ESCC are still unknown. In this study, we used high-throughput sequencing to identify circRNAs that were differentially expressed in ESCC tissues versus corresponding paracancerous tissues. Based on our findings, we discovered that circRUNX1, which is derived from RUNX1 pre-mRNA, was highly expressed in ESCC tissues and cell lines and was related to tumor differentiation grade and TNM stage. Moreover, overexpression of circRUNX1 was found to promote the proliferation and metastasis of ESCC cells in vitro and in vivo, implying that circRUNX1 might act as an oncogenic modulator. This prompted us to investigate the molecular mechanism and role of circRUNX1 in ESCC.

A growing number of studies show that N6-methyladenosine (m6A) RNA modification plays a multifaceted role in tumor formation and progression. Our previous study found that METTL3 acts as a tumor suppressor in PTC by influencing the m6A modification of c-Rel and RelA mRNAs, thereby altering tumor-associated neutrophils (TANs) infiltration to regulate tumor growth [26]. Here, we are the first to report that elevated expression of circRUNX1 in ESCC was induced by m6A RNA modification, which was accomplished by increased m6A reader IGF2BP2 level in ESCC. As a member of the IGF2BP family, IGF2BP2 has been shown to act as an mRNA stabilizer, promoting the accumulation of its target mRNAs in an m6A-dependent manner [27, 28]. Lu et al. found that IGF2BP2 could promote proliferation, migration, and invasion of ESCC cells [29], which was consistent with our results. Another novel ESCC mechanism discovered in our study was that high levels of IGF2BP2 reduced circRUNX1 degradation in an m6A-dependent manner. Further rescue experiments revealed that circRUNX1 was involved in the regulation of IGF2BP2 in terms of proliferation and metastasis of esophageal squamous cell carcinoma. To the best of our knowledge, this is the first study to provide evidence for the regulatory mechanism of m6A modification-mediated circRNA expression upregulation in ESCC. However, the specific m6A methylation sites of circRUNX1 need to be further elucidated.

Herein, we also discovered the significantly up-regulated expression of FOXP3 and the positive correlation between FOXP3 and circRUNX1 in ESCC tissues. As a major transcription factor regulatory T cell (Treg) lineage, FOXP3 plays an important role in Treg cell development and immune regulation [30, 31]. Ample evidence has confirmed that FOXP3 is not only specifically expressed in Treg cells derived from the thymus or peripheral, but also abnormally expressed in a variety of tumor cells, and is closely related to the occurrence, development, and prognosis of tumors [32–36]. Yang et al. demonstrated that FOXP3 could promote tumor growth and metastasis as well as induce EMT in non-small cell lung cancer by facilitating the Wnt/β-catenin signaling pathway [37]. In addition, Xu et al. found that FOXP3 was highly expressed in ESCC and could regulate T cell differentiation to promote immune escape [38]. However, reports of FOXP3 as a tumor-promoting factor have not been thoroughly validated. Utilizing RNA-seq analysis, we noticed that FOXP3 might act as the downstream molecule of circRUNX1 in ESCC cell lines. Moreover, qRT-PCR and immunohistochemistry were used to confirm the differential expression of FOXP3 in ESCC tissues and adjacent tissues. Additionally, in vitro functional experiments revealed FOXP3's role as a modulator of circRUNX1-mediated ESCC cell proliferation and metastasis. Furthermore, the pro-tumorigenic effects were validated in subcutaneous tumor models and lung metastasis models. Taken together, this is the first report focusing on the function of FOXP3 in ESCC cells.

Treg cells, as a subset of CD4^+^ T cells with significant immunosuppressive effects, are indispensable for maintaining systemic immune homeostasis. By suppressing antitumor immunity, Treg cells impede immune surveillance in cancer-free individuals, thereby triggering tumor immune escape [39]. Multiple cytokines such as IL-8 [40], IL-10 [41], and secreted TGF-β [42] recruit Treg cells to negatively affect immune regulation. FOXP3, as a recognized Treg cell surface marker, is an essential factor for Treg cell development and function [43]. In our study, we have identified FOXP3 as a tumor-promoting factor in ESCC. Nevertheless, further research into downstream mechanisms of FOXP3 is required. According to the current findings, we surmise that FOXP3 may promote the malignant phenotype of ESCC cells by affecting a specific signaling pathway. Another hypothesis that needs to be validated is whether FOXP3 could influence Treg cell development by recruiting inflammatory factors, thereby regulating ESCC immune escape. Furthermore, it is worth investigating whether circRUNX1 leads to the acquisition of other phenotypes in ESCC, such as tumor immunity, stemness traits or metabolism. Our research uncovered that the IGF2BP2-mediated circRUNX1/miR-449b-5p/FOXP3 axis was involved in the pathogenesis and progression of ESCC, providing a novel diagnostic marker and therapeutic target for ESCC patients.

## Conclusion

In summary, we found that circRUNX1, a novel circRNA, was up-regulated in ESCC tissues and cell lines. Moreover, circRUNX1 facilitated the proliferation and metastasis of ESCC cells in vitro and in vivo. Mechanistically, circRUNX1 acted as the sponge of miR-449b-5p to promote ESCC progression by inducing FOXP3 expression. In addition, RNA binding protein IGF2BP2 inhibited circRUNX1 degradation in an m6A-dependent manner. Collectively, our findings shed light on a new regulatory network that could aid in the development of new treatment strategies for ESCC patients.

## Supplementary Information

**Additional file 1:** **Figure S1.** High expression of circRUNX1 is related to advanced TNM stage and differentiation grade. **Figure S2.** CircRUNX1 overexpression promotes ESCC progression. **Figure S3.** FOXP3 is a downstream target of circRUNX1. **Figure S4.** FOXP3 knocking down inhibites ESCC cell proliferation and metastasis. **Figure S5.** CircRUNX1 facilitates ESCC cell progression by regulating FOXP3 in vitro. **Figure S6.** CircRUNX1 functions as a miR-449b-5p sponge in ESCC cells. **Figure S7.** The cancer-inhibiting effect of miR-449b-5p in ESCC cells can be reversed by FOXP3. **Figure S8.** Relative expression of seven candidate circRUNX1 binding proteins in ESCC tissues was predicted from GEPIA. **Figure S9.** Depletion of circRUNX1 rescues the promotive effect of IGF2BP2 overexpression on malignant behaviors in ESCC cells. **Table S1.** Sequences of siRNA and shRNA used in this study. **Table S2.** Primers used in this study. **Table S3.** Information of top 8 circRNAs in ESCC tissues. **Table S4.** The correlation between circRUNX1 and ESCC clinicopathological features. **Table S5.** The correlation between IGF2BP2 and ESCC clinicopathological features.

## Data Availability

Data generated or analyzed during this study are available from the corresponding authors on reasonable request.

## Abbreviations

ESCC: Esophageal squamous cell carcinoma
circRNA: Circular RNA
miRNA: MicroRNA
FOXP3: Forkhead box protein 3
IGF2BP2: Insulin-like growth factor 2 mRNA-binding protein 2
RUNX1: Runt-related transcription factor 1
DDHD2: DDHD domain containing 2
SLC38A1: Solute carrier family 38 member 1
PTEN: Phosphatase and tensin homolog
ceRNA: Competing endogenous RNA
qRT-PCR: Quantitative real-time polymerase chain reaction
FISH: Fluorescence in situ hybridization
IHC: Immunohistochemistry
H&E: Hematoxylin and eosin
RIP: RNA immunoprecipitation
GEPIA: Gene Expression Profiling Interactive Analysis
