# Supplementary material for: IGF2BP2-induced circRUNX1 facilitates the growth and metastasis of esophageal squamous cell carcinoma through miR-449b-5p/FOXP3 axis
Source: J Exp Clin Cancer Res. 2022 Dec 15;41:347. doi: 10.1186/s13046-022-02550-8 (PMC9753396; doi:10.1186/s13046-022-02550-8)
Supplement: Supplementary file 1 — Additional file 1: Figure S1. High expression of circRUNX1 is related to advanced TNM stage and differentiation grade. Figure S2. CircRUNX1 overexpression promotes ESCC progression. Figure S3. FOXP3 is a downstream target of circRUNX1. Figure S4. FOXP3 knocking down inhibites ESCC cell proliferation and metastasis. Figure S5. CircRUNX1 facilitates ESCC cell progression by regulating FOXP3 in vitro. Figure S6. CircRUNX1 functions as a miR-449b-5p sponge in ESCC cells. Figure S7. The cancer-inhibiting effect of miR-449b-5p in ESCC cells can be reversed by FOXP3. Figure S8. Relative expression of seven candidate circRUNX1 binding proteins in ESCC tissues was predicted from GEPIA. Figure S9. Depletion of circRUNX1 rescues the promotive effect of IGF2BP2 overexpression on malignant behaviors in ESCC cells. Table S1. Sequences of siRNA and shRNA used in this study. Table S2. Primers used in this study. Table S3. Information of top 8 circRNAs in ESCC tissues. Table S4. The correlation between circRUNX1 and ESCC clinicopathological features. Table S5. The correlation between IGF2BP2 and ESCC clinicopathological features. [file 13046_2022_2550_MOESM1_ESM.docx]

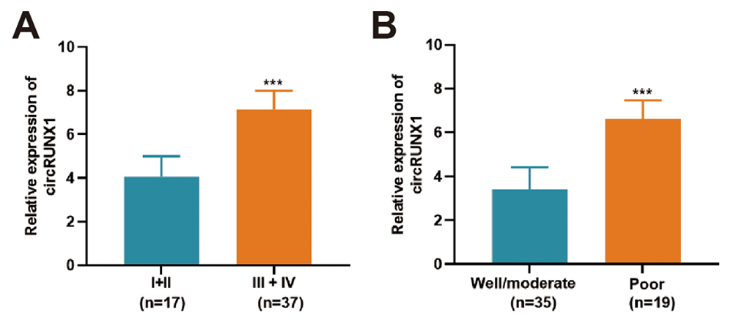


Fig. S1 High expression of circRUNX1 is related to advanced TNM stage and differentiation grade.

**A** Relative differential expression of circRUNX1 in pathological stages I+II (n=17) and III+IV (n=37) ESCC tissues was measured by qRT-PCR. **B** Relative differential level of circRUNX1 in well/moderate differentiation grade (n=35) and poor differentiation grade (n=19) ESCC tissues measured by qRT-PCR. **P* < 0.05, ***P* < 0.01, ****P* < 0.001.





Fig. S2 CircRUNX1 overexpression promotes ESCC progression.

**A** Overexpression efficiency of circRUNX1 in ESCC cells was detected by qRT-PCR. **B** The relative expression of RUNX1 mRNA was detected by qRT-PCR after circRUNX1 overexpression. **C** CCK-8 assays were performed to evaluate the cell viability after overexpressing circRUNX1. **D** The proliferation abilities of KYSE150 and TE1 cells were evaluated by colony formation assays. **E** EdU assays detected the effect of circRUNX1 overexpression on cell proliferation (Magnification × 100. Scale bar: 1 00 μm). **F** Transwell assays were performed to evaluate cell migration and invasion after overexpressing circRUNX1. **G** Migration abilities of KYSE150 and TE1 cells after overexpressing circRUNX1 were evaluated by wound healing assay. **H** Quantification of the number of colonies from colony formation assays. **P* < 0.05, ***P* < 0.01, ****P* < 0.001.


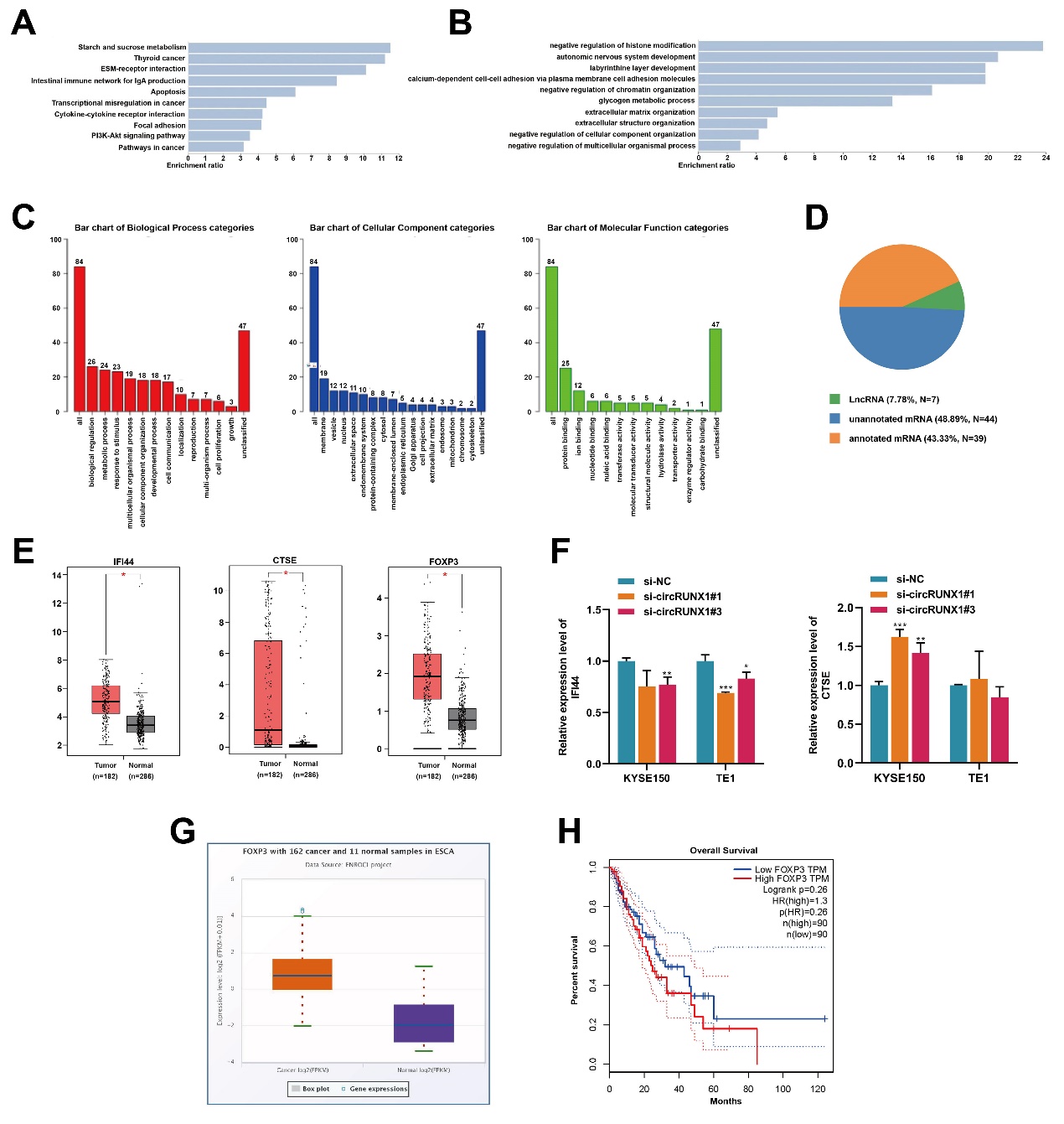


Fig. S3 FOXP3 is a downstream target of circRUNX1.

**A** and **B** KEGG pathway enrichment analysis of differentially expressed mRNAs after silencing circRUNX1. **C** GO function analysis of differentially expressed mRNAs after silencing circRUNX1. **D** Pieplot showing the classification of differentially expressed genes regulated by circRUNX1. **E** The expression of three genes in ESCC was predicted from GEPIA. **F** Relative mRNA levels of IFI44 and CTSE were detected in ESCC cells with circRUNX1 knockdown using qRT-PCR. **G** The differential expression of FOXP3 in ESCC was predicted from Targetscan. **H** The relationship between FOXP3 expression and overall survival of ESCC patients was predicted from GEPIA. **P* < 0.05, ***P* < 0.01, ****P* < 0.001.


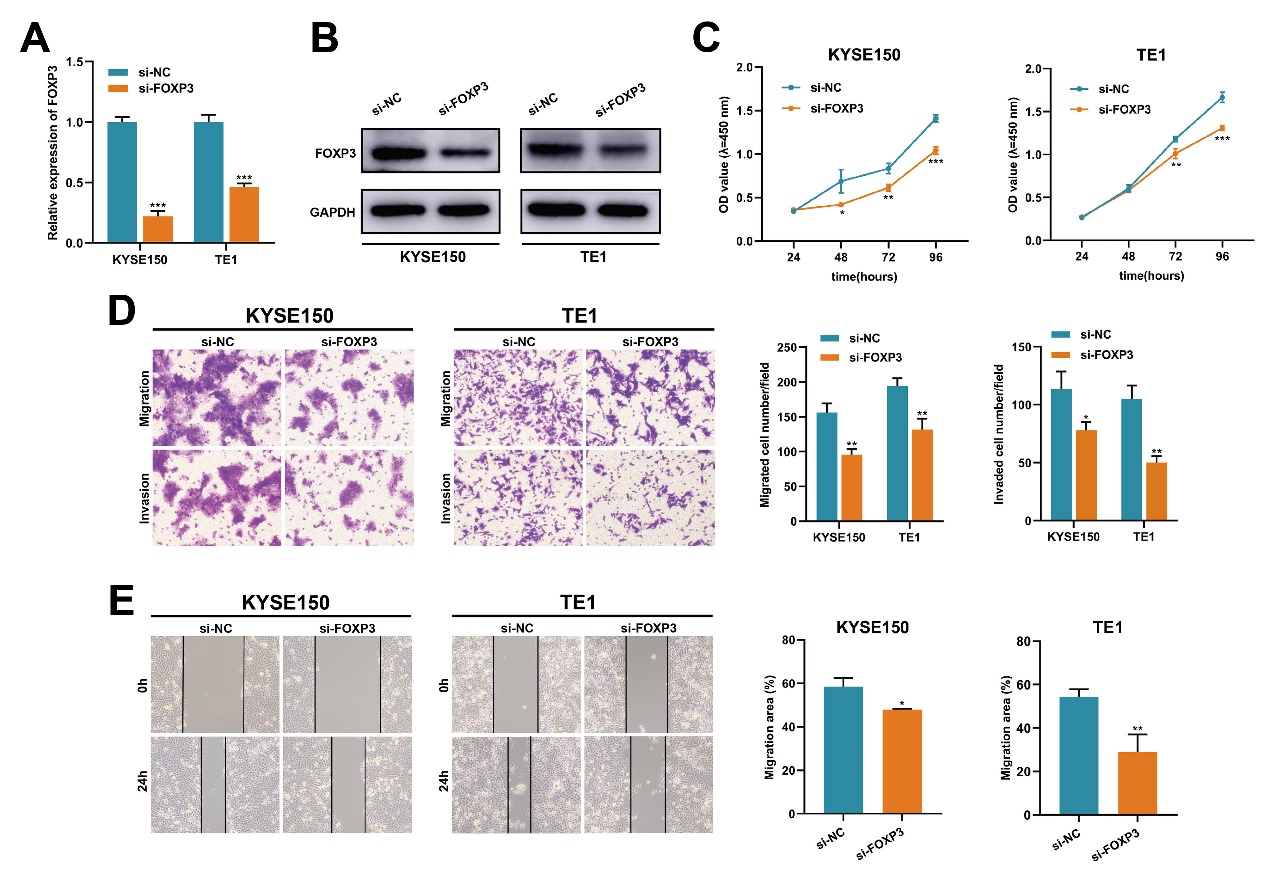


Fig. S4 FOXP3 knocking down inhibits ESCC cell proliferation and metastasis.

**A** and **B** FOXP3 knockdown efficiencies were examined in KYSE150 and TE1 cells by qRT-PCR (**A**) and western blot (**B**). **C** CCK-8 assays were performed to investigate ESCC cell proliferation ability after silencing of FOXP3. **D** and **E** Wound healing and Transwell assays were conducted to examine the role of FOXP3 in ESCC cell migration and invasion. **P* < 0.05, ***P* < 0.01, ****P* < 0.001.


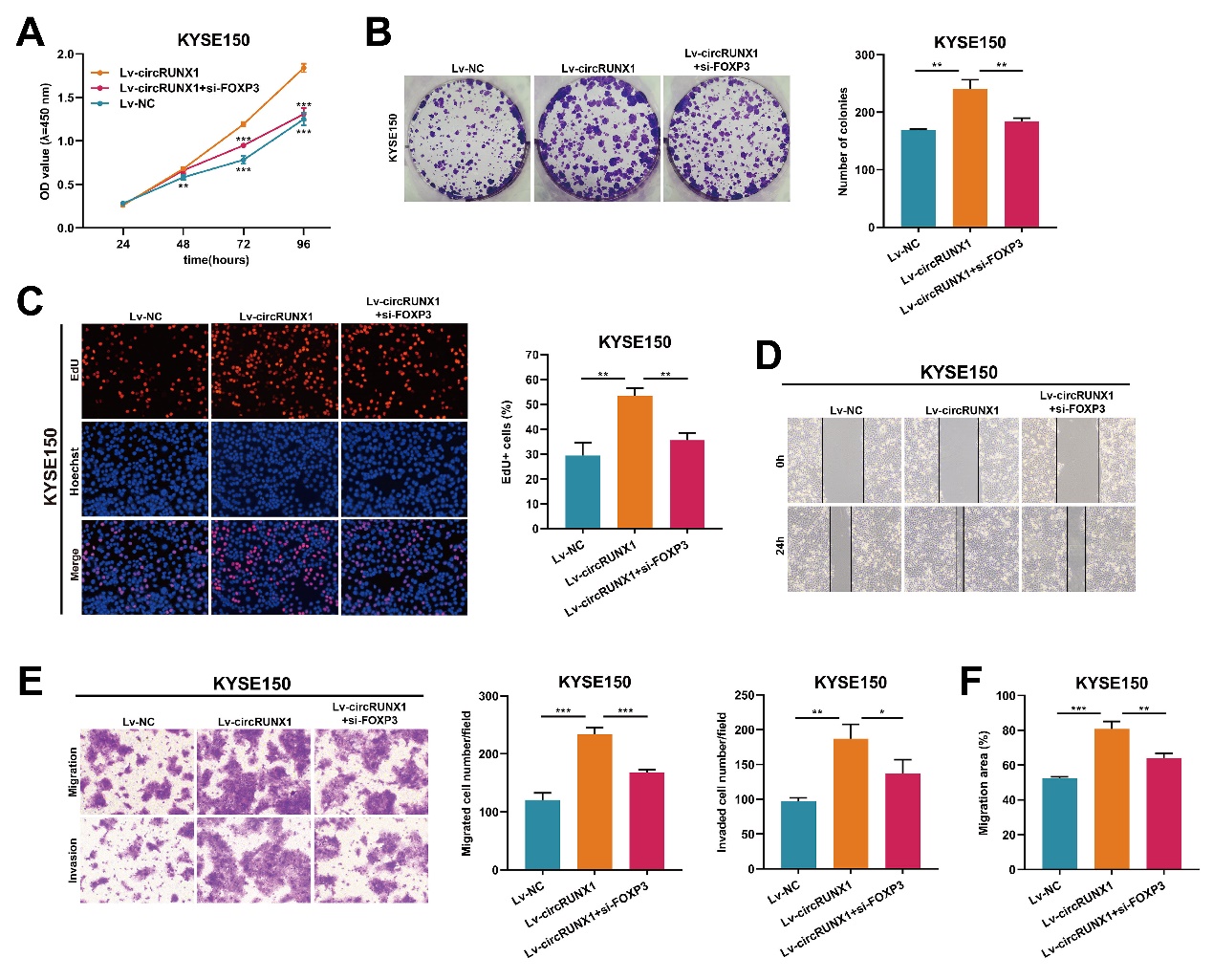


Fig. S5 CircRUNX1 facilitates ESCC cell progression by regulating FOXP3 in vitro.

**A-C** CCK-8 (**A**), colony formation (**B**), and EdU assays (**C**) were performed to determine the proliferation ability of circRUNX1 and FOXP3 in KYSE150 cells. **D-F** Wound healing and transwell assays were performed to determine the proliferation ability of circRUNX1 and FOXP3 in KYSE150 cells. **P* < 0.05, ***P* < 0.01, ****P* < 0.001.


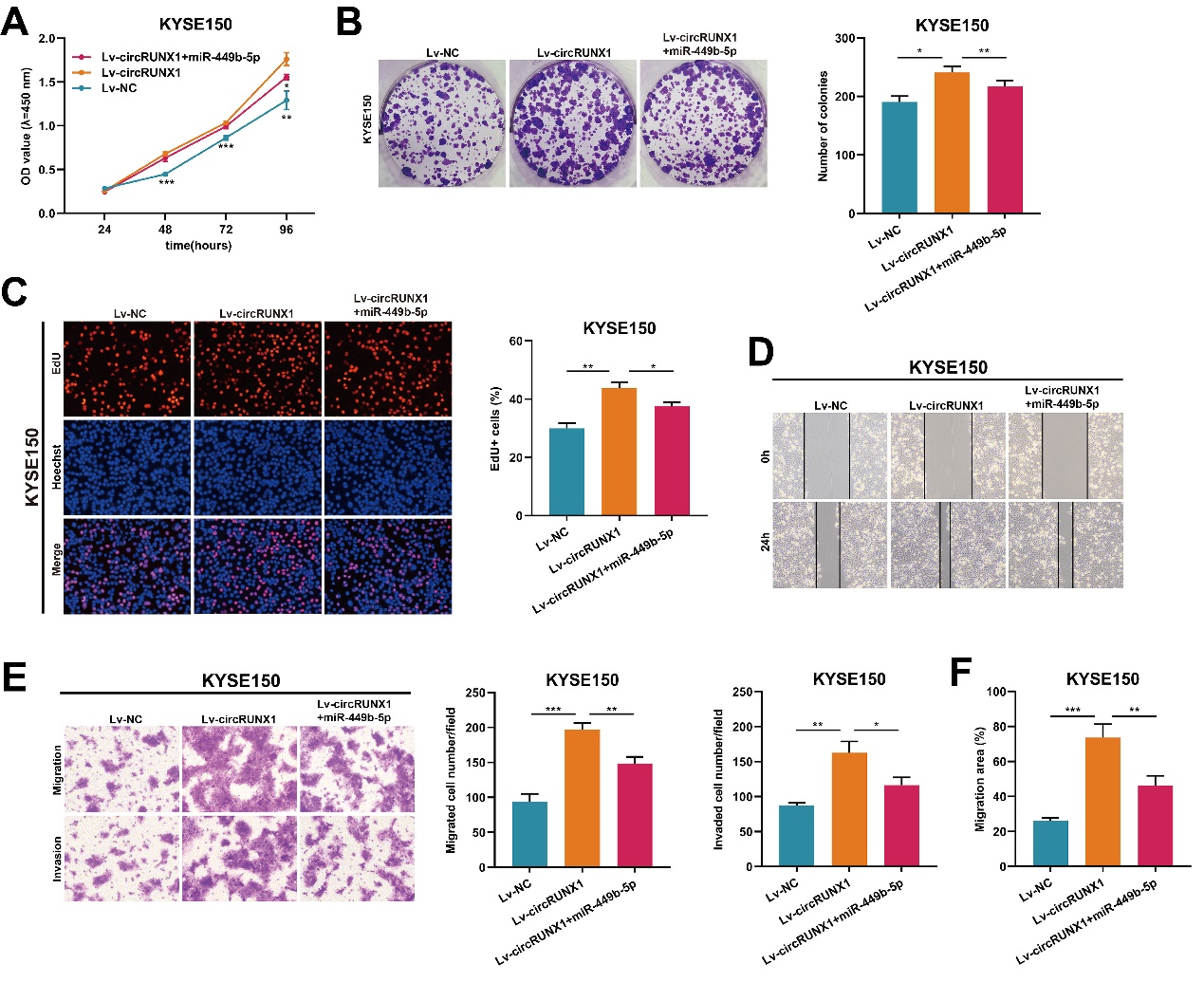


Fig. S6 CircRUNX1 functions as a miR-449b-5p sponge in ESCC cells.

**A-C** Cell proliferation was observed in CCK-8 (**A**), colony formation (**B**), and EdU assays (**C**) after transfecting miR-449b-5p mimics with circRUNX1 overexpression in KYSE150 cells. **D-F** Wound healing and transwell assays detected the migratory capacity after transfecting miR-449b-5p mimics with circRUNX1 overexpression in KYSE150 cells. **P* < 0.05, ***P* < 0.01, ****P* < 0.001.


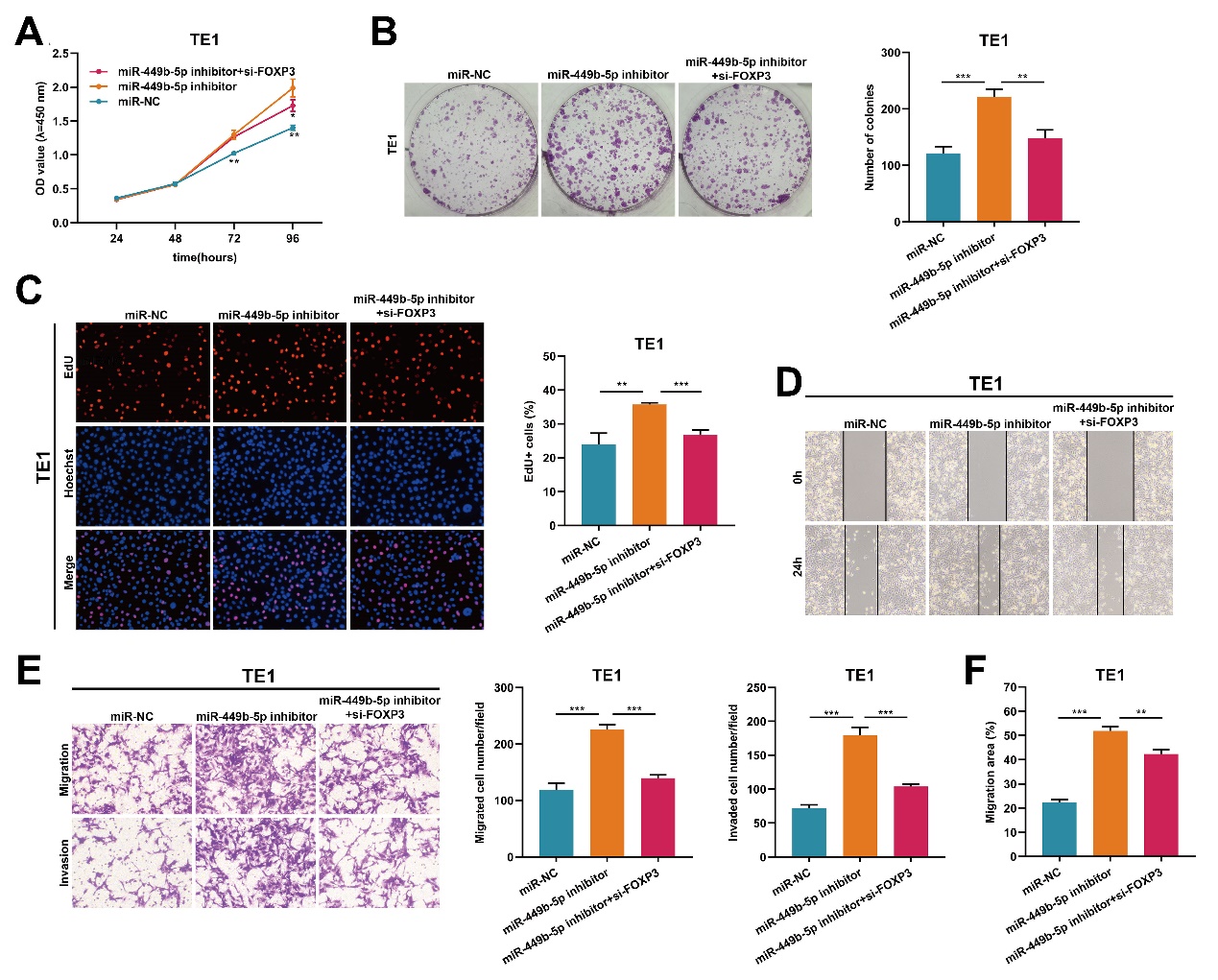


Fig. S7 The cancer-inhibiting effect of miR-449b-5p in ESCC cells can be reversed by FOXP3.

**A-C** Assessment of the proliferation of TE1 cells transfected with miR-NC, miR-449b-5p inhibitor, or cotransfected with miR-449b-5p inhibitor and si-FOXP3 by CCK-8 (**A**), colony formation (**B**), and EdU assays (**C**). **D-F** Assessment of the migration and invasion of TE1 cells transfected with miR-NC, miR-449b-5p inhibitor, or cotransfected with miR-449b-5p inhibitor and si-FOXP3 by wound healing and transwell assays. **P* < 0.05, ***P* < 0.01, ****P* < 0.001.


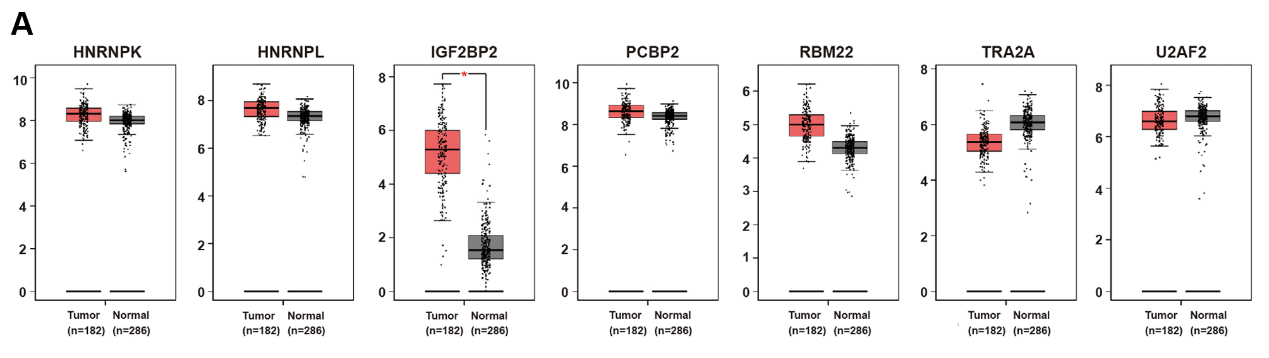


Fig. S8 **A** Relative expression of seven candidate circRUNX1 binding proteins in ESCC tissues was predicted from GEPIA.


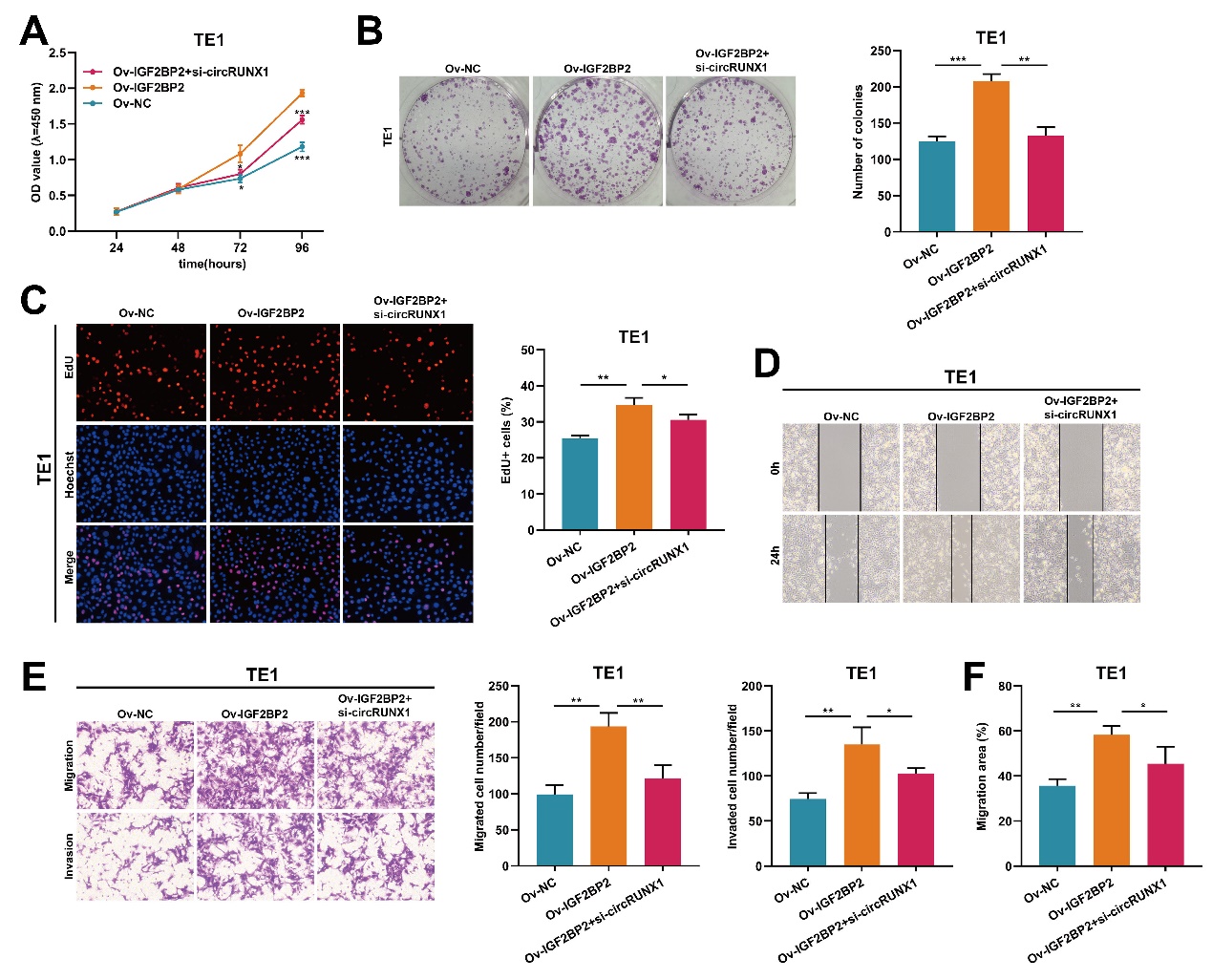


Fig. S9 Depletion of circRUNX1 rescues the promotive effect of IGF2BP2 overexpression on malignant behaviors in ESCC cells.

**A-C** The proliferation ability of TE1 cells with IGF2BP2 overexpression or IGF2BP2 overexpression plus circRUNX1 depletion was assessed by CCK-8 (**A**), colony formation (**B**), and EdU assays (**C**). **D-F** The migration and invasion abilities of TE1 cells with IGF2BP2 overexpression or IGF2BP2 overexpression plus circRUNX1 depletion were assessed by wound healing and transwell assays. **P* < 0.05, ***P* < 0.01, ****P* < 0.001.

Table S1. Sequences of siRNA and shRNA used in this study.

| Gene name | Probe (5’-3’) | |
| --- | --- | --- |
| si-circRUNX1#1 | GAGTCAGATGCAGGGGAAA | |
| si-circRUNX1#2 | GTCAGATGCAGGGGAAAAG | |
| si-circRUNX1#3 | CAGATGCAGGGGAAAAGCT | |
| si-FOXP3 | GGCTGCACCCAAAGCCTCA | |
| sh-IGF2BP2 | AGTGAAGCTGGAAGCGCATAT | |
| hsa-miR-449b-5p mimics | | AGGCAGUGUAUUGUUAGCUGGC |
| hsa-miR-449b-5p inhibitor | | AGGCAGUGUAUUGUUAGCUGGC |

Table S2. Primers used in this study.

| Name | \| Primer sequences(5'-3') \| \| --- \| |
| --- | --- | --- |
| circRUNX1 | divergent-F: AGTCAGATGCAGGGGAAAAGC |
|  | divergent-R: CTGCCGATGTCTTCGAGGTT |
|  | convergent-F: TCGGCAGAAACTAGATGATCAGA |
|  | convergent-R: ATCTGACTCTGAGGCTGAGG |
| has_circ_0000799 | F: CAGAATTGGTACCATGGGCG |
|  | R: CACCCTCTTCAACCCCTCAT |
| has_circ_0003218 | F: GGAGATCCCCAAGAGTGTCA |
|  | R: TGAGTGGTGTTGTGTCAGGA |
| has_circ_0069981 | F: CCACGTTGATGAGCGAACAA |
|  | R: GATCTTCCAGCCACAACCAC |
| has_circ_0000657 | F: GGACCAGGCCATTGTTGATC |
|  | R: AGGTGTAGGAAGACTGTGGC |
| has_circ_0005019 | F: GCTCAAGTACATGCACGACC |
|  | R: AGAAGTTCTCACCAGGCTC |
| has_circ_0001860 | F: CAGCTGTCAGATGGGTCAGA |
|  | R: TGGTGCTCTCTTCCTCTTCA |
| has_circ_0000266 | F: CCATCTGTCCCTCTCCATCC |
|  | R: TCTCTGAGAACACTGCAGCA |
| RUNX1 | F: GGAAGTCAACCTCTGCTGCT |
|  | R: CGGACCACAGAGCACTTTCT |
| FOXP3 | F: GTGGCCCGGATGTGAGAAG |
|  | R: GGAGCCCTTGTCGGATGATG |
| CTSE | F: CTGGCCCTTCCGACAAGATT |
|  | R: TCCACGAAGTCCAGTAGGGT |
| IFI44 | F: GCCTGTGCAGGGATGACATA |
|  | R: AGCCACATGTACCACACCAG |
| IGF2BP2 | F: AGCTAAGCGGGCATCAGTTTG |
|  | R: CCGCAGCGGGAAATCAATCT |
| GAPDH | F: CAGGAGGCATTGCTGATGAT |
|  | R: GAAGGCTGGGGCTCATTT |
| U6 | \| F: CTCGCTTCGGCAGCACA \| \| --- \| |
|  | \| R: AACGCTTCACGAATTTGCGT \| \| --- \| |
| miR-6751-3p | GACTGAGCCTCTCTCTCTCCAG |
| miR-762 | TAATCATATGGGGCTGGGGCC |
| miR-760 | ATATCGGCTCTGGGTCTGTGG |
| miR-7160-5p | ATATGCTGAGGTCCGGGCTGT |
| miR-8070 | CATGTGATTGACGGCTGACTCCA |
| miR-6884-5p | CGAGAGGCTGAGAAGGTGATGTTG |
| miR-4296 | CGATGTGGGCTCAGGCTCA |
| miR-6846-5p | ATATGGGGGCTGGATGGGGTA |
| miR-34c-5p | GCGAGGCAGTGTAGTTAGCTGATTG |
| mir-449a | ACGTGGCAGTGTATTGTTAGCTGGT |
| miR-449b-5p | AGGCAGTGTATTGTTAGCTGGC |
| miR-2682-5p | CAGGCAGTGACTGTTCAGACGTC |
| miR-4270 | CGATATCAGGGAGTCAGGGGAG |
| miR-6754-5p | ATACCAGGGAGGCTGGTTTGGA |

Table S3. Information of top 8 circRNAs in ESCC tissues.

| Gene name | Transcript name | Biotype | Mapped circRNA ID | Exon/intron Count | log_2_(Foldchange) | *P*-value |
| --- | --- | --- | --- | --- | --- | --- |
| [BPTF](http://www.ncbi.nlm.nih.gov/gene/?term=2186) | ENST00000321892 | circRNA | hsa_circ_0000799 | 23,24,25,26,27,28,29 | 12.222 | 2.88E-270 |
| [BMPR2](http://www.ncbi.nlm.nih.gov/gene/?term=659) | ENST00000374580 | circRNA | hsa_circ_0003218 | 2,3 | 12.144 | 8.71E-260 |
| RUNX1 | ENST00000300365 | circRNA | hsa_circ_0002360 | 5,6 | 11.781 | 5.85E-216 |
| [MTHFD2L](http://www.ncbi.nlm.nih.gov/gene/?term=441024) | ENST00000395759 | circRNA | hsa_circ_0069981 | 2,3,4,5 | 11.674 | 1.91E-204 |
| [MCTP2](http://www.ncbi.nlm.nih.gov/gene/?term=55784) | ENST00000456504 | circRNA | hsa_circ_0000657 | 2 | 11.559 | 1.14E-192 |
| [CHSY1](http://www.ncbi.nlm.nih.gov/gene/?term=22856) | ENST00000254190 | circRNA | hsa_circ_0005019 | 2 | 11.559 | 1.14E-192 |
| [ZCCHC7](http://www.ncbi.nlm.nih.gov/gene/?term=84186) | ENST00000336755 | circRNA | hsa_circ_0001860 | 2 | 11.433 | 1.33E-180 |
| [FAM53B](http://www.ncbi.nlm.nih.gov/gene/?term=9679) | ENST00000494792 | ciRNA | novel_circ_0000266 | 6 | 11.433 | 1.33E-180 |

ciRNA: circulated by intron, circRNA: circulated by exon

Table S4. The correlation between circRUNX1 and ESCC clinicopathological features.

|  | CircRUNX1 expression | | *P*-value |
| --- | --- | --- | --- |
|  | High (n=27) | Low (n=27) |  |
| **Gender** |  |  |  |
| Male | 17 | 19 | 0.7734 |
| Female | 10 | 8 |  |
| **Age** |  |  |  |
| ≤ 65 years | 6 | 12 | 0.1480 |
| > 65 years | 21 | 15 |  |
| **Tumor size** |  |  |  |
| ≤ 3 cm | 12 | 9 | 0.5772 |
| > 3 cm | 15 | 18 |  |
| **Differentiation grade** |  |  |  |
| Well+Moderate | 13 | 22 | **0.0214*** |
| Poor | 14 | 5 |  |
| **TNM stage** |  |  |  |
| I+II | 4 | 13 | **0.0177*** |
| III + IV | 23 | 14 |  |
| **Lymphatic metastasis** |  |  |  |
| Absent | 11 | 14 | 0.5857 |
| Present | 16 | 13 |  |

Table S5. The correlation between IGF2BP2 and ESCC clinicopathological features.

|  | IGF2BP2 expression | | *P*-value |
| --- | --- | --- | --- |
|  | High (n=21) | Low (n=20) |  |
| **Gender** |  |  |  |
| Male | 14 | 14 | >0.9999 |
| Female | 7 | 6 |  |
| **Age** |  |  |  |
| ≤ 65 years | 6 | 9 | 0.3048 |
| > 65 years | 15 | 11 |  |
| **Tumor size** |  |  |  |
| ≤ 3 cm | 7 | 11 | 0.2146 |
| > 3 cm | 14 | 9 |  |
| **Differentiation grade** |  |  |  |
| Well+Moderate | 15 | 12 | 0.5204 |
| Poor | 6 | 8 |  |
| **TNM stage** |  |  |  |
| I+II | 4 | 5 | >0.9999 |
| III + IV | 17 | 15 |  |
| **Lymphatic metastasis** |  |  |  |
| Absent | 5 | 12 | **0.0278*** |
| Present | 16 | 8 |  |
